# Spatial cluster detection using dynamic programming

**DOI:** 10.1186/1472-6947-12-22

**Published:** 2012-03-25

**Authors:** Yuriy Sverchkov, Xia Jiang, Gregory F Cooper

**Affiliations:** 1 Intelligent Systems Program, University of Pittsburgh, Pittsburgh, PA, USA; 2 Department of Biomedical Informatics, University of Pittsburgh, Pittsburgh, PA, USA

## Abstract

**Background:**

The task of spatial cluster detection involves finding spatial regions where some property deviates from the norm or the expected value. In a probabilistic setting this task can be expressed as finding a region where some event is significantly more likely than usual. Spatial cluster detection is of interest in fields such as biosurveillance, mining of astronomical data, military surveillance, and analysis of fMRI images. In almost all such applications we are interested both in the question of whether a cluster exists in the data, and if it exists, we are interested in finding the most accurate characterization of the cluster.

**Methods:**

We present a general dynamic programming algorithm for grid-based spatial cluster detection. The algorithm can be used for both Bayesian maximum *a-posteriori *(MAP) estimation of the most likely spatial distribution of clusters and Bayesian model averaging over a large space of spatial cluster distributions to compute the posterior probability of an unusual spatial clustering. The algorithm is explained and evaluated in the context of a biosurveillance application, specifically the detection and identification of Influenza outbreaks based on emergency department visits. A relatively simple underlying model is constructed for the purpose of evaluating the algorithm, and the algorithm is evaluated using the model and semi-synthetic test data.

**Results:**

When compared to baseline methods, tests indicate that the new algorithm can improve MAP estimates under certain conditions: the greedy algorithm we compared our method to was found to be more sensitive to smaller outbreaks, while as the size of the outbreaks increases, in terms of area affected and proportion of individuals affected, our method overtakes the greedy algorithm in spatial precision and recall. The new algorithm performs on-par with baseline methods in the task of Bayesian model averaging.

**Conclusions:**

We conclude that the dynamic programming algorithm performs on-par with other available methods for spatial cluster detection and point to its low computational cost and extendability as advantages in favor of further research and use of the algorithm.

## Background

The task of spatial cluster detection involves finding spatial regions where some property deviates from the norm or the expected value. In a probabilistic setting this task can be expressed as finding a region where some event is significantly more likely than usual. Spatial cluster detection is of interest in fields such as biosurveillance, mining of astronomical data, military surveillance [1], and analysis of fMRI images [2]. A well-known early method for spatial cluster detection is the spatial scan statistic developed by Kulldorff [3]. This approach is formulated as a frequentist method for cluster detection and can be summarized as the process of maximizing the likelihood ratio statistic

(1) $$F\left(S\right)=\frac{P\left(\text{Data}|H_{1}\left(S\right)\right)}{P\left(\text{Data}|H_{0}\right)}$$

for each subregion of interest *S *and computing the statistical significance of the statistic using randomization testing. Here *H*_0 _represents the null hypothesis that no clusters (e.g., disease outbreak regions) are present, and *H*_1_(*S*) represents the alternative hypothesis that subregion *S *is the location of a cluster. While the original paper [3] is restricted to scanning for a single circular cluster, the method has been extended to ovals and other shapes as well as to space-time statistics in later works [4-10].

One of the main drawbacks of Kulldroff's approach is the computational expense associated with the randomization testing required to calculate statistical significance. Neill and Moore [11] address this problem by developing a method of cleverly reducing the number of different possible subregions considered. While the cost of randomization testing of each hypothesis stays the same, reducing the number of hypotheses to consider reduces the overall computational cost significantly. Their method makes use of an *overlap-kd tree *data structure to apply bounds on *F(S) *to prune the search. This method was later extended to multiple dimensions in [12].

Another approach taken in the literature is the development of Bayesian variants on Kulldroff's statistic [13,14]. The Bayesian approach replaces the likelihood ratio with a posterior probability

(2) $$P\left(H_{1}\left(S\right)|\text{Data}\right)=\frac{P\left(H_{1}\left(S\right)\right)P\left(\text{Data}|H_{1}\left(S\right)\right)}{P\left(\text{Data}\right)}$$

The use of a Bayesian approach eliminates the need for significance testing since the purpose of significance testing is to measure how likely the alternative hypothesis is given the data (more precisely, it tells us how likely we are to obtain a likelihood ratio that is at least as extreme under the null hypothesis). Since the posterior probability gives us the probability of the alternative hypothesis given the data directly, the need for time-consuming randomization testing is eliminated. An additional benefit of the Bayesian approach is that the introduction of prior probabilities enables these methods to incorporate prior information to potentially enhance detection [13,15]. In some cases, however, the task of selecting useful priors can prove challenging.

The methods mentioned above so far are "count-based" methods which detect clusters based on aggregate counts of data such as the total number of ED visits in a given ZIP code on a given day, for example. An alternative approach is "agent-based," where each individual in the population is modeled. This is the approach taken by Cooper et al. [16] in developing a disease surveillance system called PANDA. The work of Jiang et al. [17] extends PANDA by incorporating spatial information, resulting in an agent-based Bayesian scan statistic. The disease model used in our work is also agent-based and can be viewed as a simplified version of the model used by PANDA. It should be noted that unlike the disease model that we use to illustrate this algorithm, PANDA and its extensions model multiple diseases and are designed to differentiate between outbreaks of the different diseases modeled.

A common way of articulating the spatial scan problem is one that considers the surveillance region to be a rectangular *R *× *C *grid of cells. The methods that use this approach, among which are [7,11-13,17,18], are sometimes referred to as grid-based methods. With this representation every subset of cells is a possible cluster *S*, or in the context of outbreak detection, a potential outbreak region.

Most grid-based methods have mainly focused on the detection of single rectangular clusters aligned with the grid.

However, since an outbreak may take other shapes, or may occur in multiple disconnected regions of the surveillance grid, it is of interest to develop algorithms that are able to detect more general subregions. Jiang and Cooper [18] developed a recursive algorithm that operates by detecting the most likely rectangular subregion that contains a cluster and refines it through a combination of unions with additional rectangular subregions as well as refined scanning within each rectangle. While this approach improves the detection of non-rectangular subregions it comes at the cost of repeatedly scanning the same region multiple times as the recursion occurs.

Complementary to these approaches are methods for calculating the posterior probability of the presence of clusters anywhere in the region using Bayesian model averaging, such as the work of Shen et al. [19]. They present a method that is sensitive to the presence of irregularly shaped or sparsely distributed outbreaks but does not favor spatially grouped clusters since individual cells are considered independently.

The task of detecting irregularly shaped clusters has been addressed by distance-based methods which when, given a set of points in (possibly many-dimensional) space find clusters of elevated or uniform density [20,21]. While these methods may be considered more suitable than grid-based methods for certain domains, they are not addressed in the current paper, which aims to build on previous grid-based methods.

We implement, develop, and test an algorithm for finding multiple rectangular clusters on a grid that employs dynamic programming in order to consider an exponential number of hypotheses in polynomial time. The algorithm finds a hypothesis *H*_1_(*S_i_*) which is most probable in the Bayesian sense given the data.

We also present an adaptation of the algorithm that averages over all considered hypotheses *H*_1_(*S_i_*) to obtain a posterior probability for the presence of a cluster anywhere in the surveillance region.

## Methods

Below, we describe and investigate an algorithm for the detection of multiple rectangular spatial clusters. The algorithm is applied to the simple outbreak model described next. While we restrict the analysis to this particular model, the algorithm is general and can be applied to any underlying model that can provide a likelihood function such as the function *lik *described in Equation (8) below. We then describe the scanning algorithm itself.

### Outbreak model

**Figure 1:**
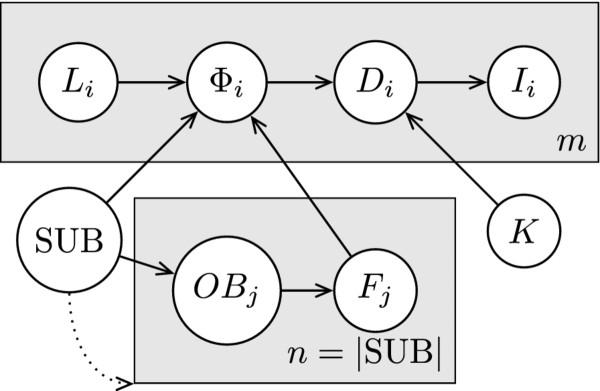
**Simple disease model**. A plate representation of the simplified entity-based model used.

We define the disease model as a Bayesian Network (BN) model, which is a type of graphical model for representing joint probability distributions; it is particularly suited for modeling conditional independence between variables. In the graphical representation each node represents a random variable and the distribution of each variable is defined as a conditional distribution that is conditioned on the variables from which it receives incoming edges, or as a prior probability distribution if the node has no incoming edges [22]. An extension to the BN representation employs plates when subgraphs of the network are replicated many times. In the graphical plate representation, plates are shown as boxes, the nodes and edges that are on each plate are replicated the number of times shown on the plate, replicated nodes are indexed, and edges that cross plate boundaries are also replicated [23]. In particular, in Figure 1 we have *m *copies of nodes *L_i_*, *D_i_*, and *I_i_*. We further extend the notation by allowing the number of replications to depend on random variables, as represented by the dotted arrow from SUB to the plate indexed by *j*. Here, the values that SUB takes are ordered sets and the number of replications *n *is defined to equal the size of the set |*SUB*|. The model is explained in further detail below, where we first describe the model and the meanings of the variables in general terms, and then provide the particular parameterization that we used to instantiate the variables in our tests.

#### Model definition and likelihood function

We employ an agent-based model where each cell of the *R *× *C *grid contains a number of individuals. The entire population, made up of *m *individuals, is modeled.

Figure 1 shows a BN plate representation of the model that governs the state of each individual: SUB = (*rect*_1_, ..., *rect_n_*) represents the (possibly disconnected) hypothesized location of an ongoing outbreak as an ordered collection of *n *non-intersecting rectangles, *OB_j _*are binary variables each of which determines whether an outbreak is present in a given rectangle indexed by *j*, and *F_j _*∈ [0,1] represent frequencies of outbreak disease cases per day, each corresponding to an outbreak rectangle indexed by *j*. We will use the vector notation shorthand **F **= (*F*_1_, ..., *F_n_*) where convenient. *K *∈ [0,1] represents the proportion of the population that visits the ED per day in the absence of an outbreak, *D_i _*represents the disease state of a given individual, *I_i _*represents the observable evidence about the individual, *L_i _*∈ 1, ..., *R*} × {1, ..., *C*} represents the grid cell in which the individual is located, and *Φ_i _*is the manifested outbreak frequency, which is the frequency of the outbreak disease per day in the individual's grid cell. The dotted arrow pointing from SUB to the plate containing *OB_j _*and *F_j _*is meant to make explicit that the number of indexes *j*, and hence copies of these nodes, is dependent on the size of SUB.

The random variable *Φ_i_*, which represents the outbreak frequency manifested in individual *i*'s grid cell, is not used to capture uncertainty as random variables normally do, but rather plays the role of a logic switch. It is defined as follows:

(3) $$\text{If}\;\exists rect_{j}\in \text{SUB}:L_{i}\in rect_{j},\;\text{then}\;P\left(\Phi_{i}=F_{j}|L_{i},\text{SUB},F\right)=1$$

(4) $$\text{If}\;∄rect_{j}\in \text{SUB}:L_{i}\in rect_{j},\;\text{then}\;P\left(\Phi_{i}=0|L_{i},\text{SUB},F\right)=1$$

Note that this is a well-defined probability distribution only under the constraint that the members of SUB do not intersect. We could alternatively merge this logic into the definition of *D_i_*, however, this decomposition of the model yields a clearer presentation.

The presence of *OB_j _*as separate from *F_j _*serves a similar logical purpose. The distribution of *OB_j _*will depend on the hypothesis space and is left for the model instantiation section. The distribution of *F_j _*is defined below in terms of *OB_j _*and a "template" frequency distribution *F *that is also a detail of model instantiation.

(5) $$P\left(F_{j}=0|OB_{j}=\text{false}\right)=1$$

(6) $$\left(F_{j}|OB_{j}=\text{true}\right)\sim F$$

Where (*F_j_*|OB*_j _*= *true*) ~ *F *indicates that *F_j _*is i.i.d. according to the distribution of *F *when *OB_j _*= true.

The primary purpose of the model is to enable us to calculate the likelihood of the presence of an outbreak in a given subregion, where a subregion is taken to be any set of cells. Throughout this paper, we will often also refer to rectangular subregions, or simply rectangles, to make explicit instances where we require the sets of cells to form multi-cell rectangles on the grid. We will also introduce below the concept of tilings and tiles. In the context of this paper, tiles are always rectangular subregions.

The data likelihood of the presence or absence of an outbreak in a subregion is given by the likelihood of the data about individuals located within the subregion's limits supporting a uniform outbreak of some frequency *f *from the frequency distribution *F *or the absence of an outbreak, respectively. In order to arrive at the likelihood of an outbreak, consider the case of calculating the likelihood of a particular observation *I_i _*given a manifested outbreak frequency *Φ_i _*and a particular *K*:

(7) $$P\left(I_{i}|\Phi_{i}\right)=\underset{D_{i}}{\sum }\;P\left(D_{i}|\Phi_{i},K\right)P\left(I_{i}|D_{i}\right)$$

Note that the likelihood for the individual given the absence of an outbreak can be obtained from the expression above by letting *Φ_i _*= 0. To obtain the likelihood of an outbreak state (not just a particular outbreak frequency) in a subregion of the grid *S*, we obtain a likelihood for each frequency by taking the product over all per-person likelihoods and take an expectation over those likelihoods to get the expected likelihood of the desired outbreak state. Formally, we will use the variable *x *to represent the outbreak state. Let *x *= 0 indicate that a subregion *S *contains no outbreak, *x *= 1 indicate that *S *is an outbreak subregion, and let the notation E_*K,F *_represent the expectation over the random variables *K *and *F*. Then the likelihood for any set of grid cells *S *under an outbreak state *x *is given by:

(8) $$lik\left(x,S\right)=E_{K,F}\;\underset{i|L_{i}\in S}{\prod }\;P\left(I_{i}|\Phi_{i}=F\cdot x\right)=E_{K,F}\;\underset{i|L_{i}\in S}{\prod }\;\underset{D_{i}}{\sum }\;P\left(D_{i}|\Phi_{i}=x\cdot F,K\right)P\left(I_{i}|D_{i}\right)$$

Here we are simplifying some of the calculation by capturing the logic behind determining the manifested frequency *Φ_i_*. Particularly, we are using the fact that the manifested outbreak frequency in a non-outbreak cell is 0, which is the end result of multiplying *x *by *F *when *x *= 0 to indicate the absence of an outbreak.

#### Model instantiation

Three disease states are modeled: noED, flu, and other, which respectively represent the events that an individual did not come in to the ED, came in to the ED due to influenza, or came to the ED for some other reason. The probability that an individual *i *comes in to the ED due to influenza is equal to the manifested outbreak frequency *Φ_i_*. Those individuals that do not come in to the ED due to influenza have a probability *K *of coming in to the ED for some other reason (e.g. due to acute appendicitis). Under this model the distribution of *D_i _*is defined by:

(9) $$P\left(D_{i}|\Phi_{i},K\right)=\left\{\begin{array}{c} \Phi_{i}\;\text{for}\;D_{i}=\text{flu}\\ \left(1-\Phi_{i}\right)K\;\text{for}\;D_{i}=\text{other}\\ \left(1-\Phi_{i}\right)\left(1-K\right)\;\text{for}\;D_{i}=\text{noED} \end{array}\right)$$

In the initial stages of the investigation *K *was treated as a constant value, i.e., a distribution with probability mass 1 at *K *= 3.904 × 10^-4 ^which is an estimate that comes from data obtained for ED visits in Allegheny County, Pennsylvania in May of the years 2003-2005. We also consider a model where *K *is a discrete distribution with positive mass at multiple values estimated from that data. However, due to practical limitations mentioned in our discussion of additional tests below, most tests were performed with the model that uses a constant *K*.

To estimate the distribution *F *that ultimately governs the distributions of outbreak frequencies in the model, we base it on a distribution previously used for a similar, more complex disease model in [17]. We adjusted the distribution to reflect a uniform density over the interval (0, 6.50 × 10^-4^].

Four evidence states are modeled for the evidence variable *I_i_*, in the form of chief complaints, taking the values cough, fever, other, and missing. *I_i _*is governed by the following distribution:

(10) $$P\left(I_{i}=\text{cough}|D_{i}=\text{flu}\right)=0.335$$

(11) $$P\left(I_{i}=\text{fever}|D_{i}=\text{flu}\right)=0.4$$

(12) $$P\left(I_{i}=\text{other}|D_{i}=\text{flu}\right)=0.265$$

(13) $$P\left(I_{i}=\text{cough}|D_{i}=\text{other}\right)=0.025$$

(14) $$P\left(I_{i}=\text{fever}|D_{i}=\text{other}\right)=0.036$$

(15) $$P\left(I_{i}=\text{other}|D_{i}=\text{other}\right)=0.939$$

(16) $$P\left(I_{i}=\text{missing}|D_{i}\neq \text{noED}\right)=0$$

(17) $$P\left(I_{i}=\text{missing}|D_{i}=\text{noED}\right)=1$$

A value of "missing" indicates that individual *i *did not come in to the ED. The values that *I_i _*takes for individuals that did come in to the ED (either due to influenza or other reasons) are governed by a probability distribution that is based on expert assessments that are informed by the medical literature.

The proper choice of prior distributions is closely tied to the proper choice of the prior probability of an influenza outbreak being present anywhere in the region. For that purpose, we take the value *P(*outbreak) = 0.04 from previous work [17,24]. This value will appear at multiple points throughout this paper.

### The distributions of the subregion, outbreak, and frequency variables

The distributions of SUB, *OB_j_*, and *F_j _*are inherently tied together, as made explicit in the BN structure, and since the space of SUB is the space of considered outbreak subregions, it is different for different algorithms that consider different hypothesis spaces. This section describes the distributions of SUB and *F_j _*for the two baseline methods we use in our evaluation and for the dynamic programming algorithm.

#### The single-rectangle case

The simplest case is the case where only single-rectangle hypotheses are considered. Under that model, SUB can represent any of the *R*(*R *+ 1)*C*(*C *+ 1)/4 rectangles that can be placed on the *R *× *C *grid, each representing a hypothesis of an outbreak within the rectangle and no outbreak outside the rectangle. SUB can take an additional value to represent a non-outbreak hypothesis. Since we defined SUB to be an ordered set of rectangles, formally, each single-rectangle hypothesis is represented as a single-element set, and the non-outbreak hypothesis is represented by the empty set ∅. The associated prior distribution of SUB is:

(18) P(SUB=∅)=1-P(outbreak)

(19) $$P\left(\text{SUB}=\left(S\right)\right)=\frac{4}{R\left(R+1\right)C\left(C+1\right)}P\left(\text{outbreak}\right)\;\text{For}\;\text{any}\;\text{rectangle}\;S$$

Note that when SUB is the empty set, *OB_j _*and *F_j _*do not need to appear in the BN as they play no role in the distribution of *Φ_i _*and the rest of the BN. In the case when SUB represents a rectangle, it is a single-element set, and there is only one *j*, namely *j *= 1. *OB*_1 _is then taken to be true and consequently *F*_1 _~ *F*.

#### The multi-rectangle case

The case that corresponds to the greedy algorithm to which we will be comparing our algorithm is the case where each element *sub *of the hypothesis space is an ordered set of non-overlapping rectangles. Call this hypothesis space SUB, that is, SUB is a set of values that the random variable SUB can take, where each value *sub *is an ordered set of rectangles. Here the prior distribution of SUB is governed by a structure prior parameter *q *as follows:

(20) $$P\left(\text{SUB}=\left(rect_{1},\ldots ,rect_{n}\right)\right)=\frac{q^{n}}{Z_{M}}\text{For}\;n>0$$

(21) $$P\left(\text{SUB}=\emptyset \right)=\frac{\alpha }{Z_{M}}$$

The parameter *α *can be used to adjust the prior probability of the absence of an outbreak, while the parameter *q *controls the relative prior probability of having a more complex outbreak hypothesis (that is, a hypothesis with more hypothesized rectangles) vs. a less complex outbreak hypothesis. *Z_M _*is a normalization constant described by:

(22) $$Z_{M}=\alpha +\underset{sub\in SUB\backslash \{\emptyset \}}{\sum }\;q^{\left|sub\right|}$$

Where \ is the set difference operator and |*sub*| denotes the size of the ordered set *sub *(a particular value that SUB can take) in terms of the number of rectangles in the set.

Since in our experiments this model is used only in the context of selecting the most likely outbreak hypothesis, there is no need to specify *α *or calculate *Z_M_*. The value *q *= *P*(*outbreak*) × 4/(*R*(*R *+ 1)*C*(*C *+ 1)) was used for the structure prior parameter.

Since in this model every rectangle that appears in SUB is an outbreak rectangle, we again have *OB_j _*= true for all *j *and consequently *F_j _*~ *F *are i.i.d., one per rectangle.

#### The tiling case

The hypothesis space used by the dynamic programming algorithm, the centerpiece of this work, is a space of tilings (discussed in more detail in the next section.) To express this hypothesis space, each value of SUB is a tiling: a collection of non-overlapping rectangles such that every cell of the grid is covered by exactly one of these rectangles. In order to represent an outbreak hypothesis as a tiling we use the variable *OB_j _*to indicate whether a tile is hypothesized to represent an outbreak or an outbreak-free region. In the tiling context the distribution over the variables SUB and *OB_j _*is defined as follows:

(23) $$P\left(\text{SUB}\right)=\frac{1}{Z_{T}}\;\text{For}\;\text{every}\;\text{tiling}\;\text{SUB}$$

(24) $$P\left(OB_{j}=\text{true}|\text{SUB}\right)=p\;\text{For}\;\text{every}\;\text{tiling}\;\text{SUB}$$

(25) $$P\left(OB_{j}=\text{false}|\text{SUB}\right)=1-p\;\text{For}\;\text{every}\;\text{tiling}\;\text{SUB}$$

Here *Z_T _*is a normalization constant and *p *is a structure prior representing the conditional probability that the rectangle *rect_j _*∈ *sub *(where *sub *is a value in the range of the r.v. SUB) is an outbreak rectangle given that it is indeed a rectangle of the hypothesis. The prior distribution of SUB is taken to be uniform since we assume that we have no information that leads us to favor one tiling over another. The calculation of *Z_T _*is left for a later section that also discusses the relationship between *p *and the prior probability of an outbreak, as well as the process which was used to select the value of *p *that corresponds to the prior probability of a flu outbreak equaling *P(*outbreak) = 0.04.

In the text that follows, we will refer to the choice of a set of tiles *sub *and the associated assignment of the variables *OB*_1_, ..., *OB_n _*as a colored tiling, or when it is clear from the context we may refer to colored tilings simply as tilings. We also discuss the particular space of colored tilings that the dynamic programming algorithm considers in more depth, since this space is a subset of the space of all possible tilings. The possibility of using a structure prior *P*(*OB_j _*= true|SUB) that is not identical for every rectangle in every tiling SUB is also discussed.

### Tilings

**Figure 2:**
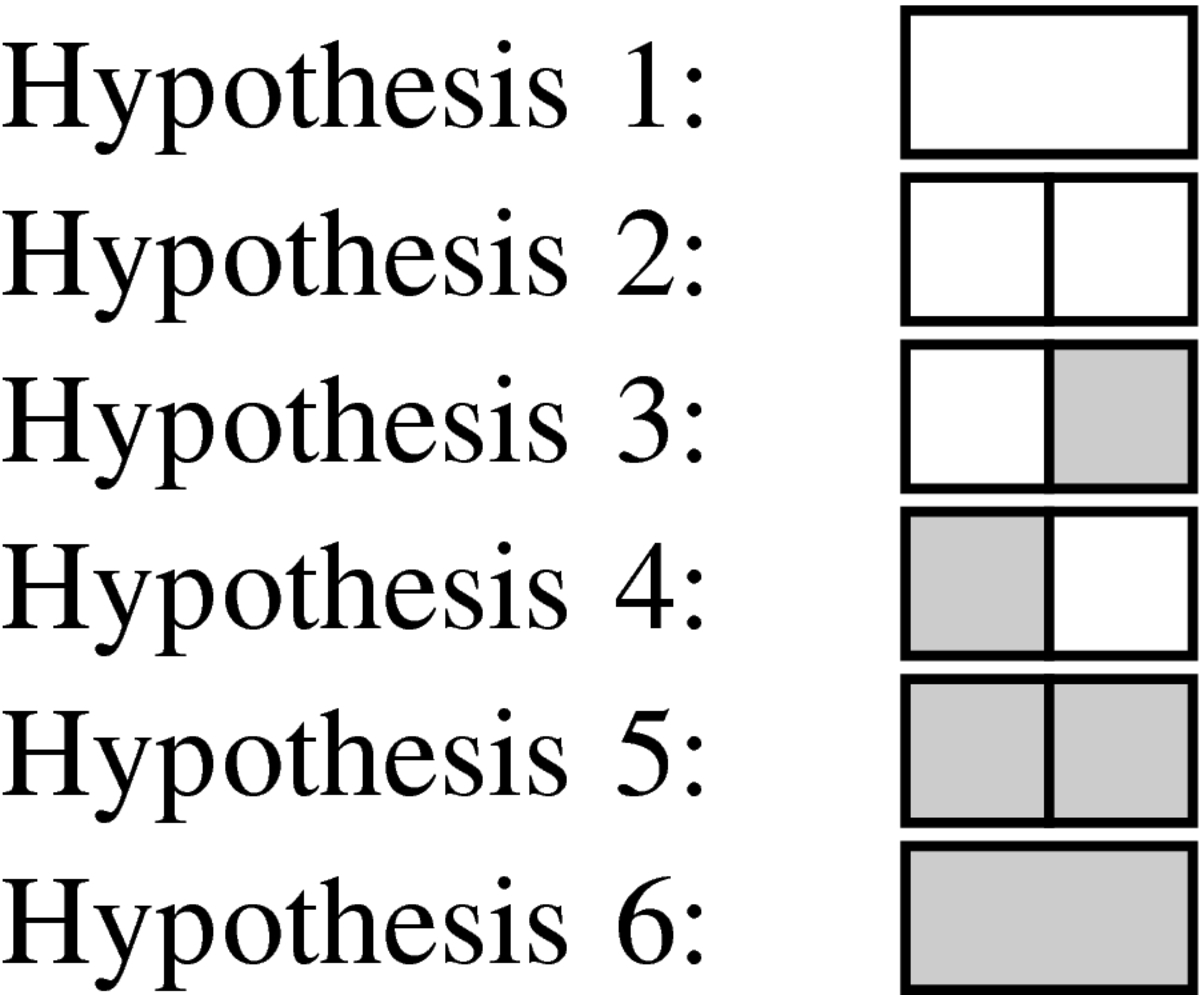
**1 by 2 colored tilings The 6 possible tilings of a 1 × 2 grid**. White tiles represent hypothesized non-outbreak regions and gray tiles represent hypothesized outbreak regions.

The algorithm presented in this paper searches a space of hypotheses where each hypothesis is a possible tiling of the surveillance grid by non-outbreak and outbreak rectangles. For example, Figure 2 shows the six possible tilings of a 1 × 2 grid.

Note that we make a distinction between two adjacent outbreak tiles (e.g., Hypothesis 5) and one large outbreak tile (e.g., Hypothesis 6). The rationale for this is that each tile represents a region over which the outbreak frequency is uniform. In the 1 × 2 example, we would expect Hypothesis 5 to be more likely than Hypothesis 6 in a case where, for example, 5% of the population in the left cell has the outbreak disease and 10% of the population in the right cell has the outbreak disease. Conversely, if the outbreak disease cases are distributed uniformly among the two cells we would expect Hypothesis 6 to be more likely.

#### Computing tiling scores

In computing tiling scores, we assume conditional independence among separate tiles given a particular tiling, even if the tiles are adjacent. As an alternative, modeling dependencies could help in the cluster detection task to adjust the prior probability of the presence of a disease in one cluster when it is near another cluster. A model that takes such effects into account could achieve improved outbreak detection, especially when modeling infectious diseases like influenza where being near an infected individual increases the probability of transmission of an infection. Modeling such dependencies incorrectly, however, could also hinder detection. In this sense, our choice not to model spatial dependencies can be seen as a cautious approach to avoid making informative spatial dependence assumptions that may be incorrect and therefore deleterious to outbreak detection performance. Even so, our basic choice of priors does favor finding a smaller number of tiles for a region, which often leads to spatially grouping neighboring disease cases.

The independence assumptions we make enable the dramatic computational efficiency that we gain by using dynamic programming, as explained in detail below. Assuming conditional independence does not constrain the outbreak hypotheses that can be identified in principle from the data, if given enough data. Although valid assumptions of conditional dependence may yield a more accurate performance in light of available data, representing and reasoning with conditional dependence carries an enormous computational burden that we avoid. It may be possible to extend our method to take spatial dependencies into account and still maintain computational tractability. We leave this issue as an open problem for future research.

Conditional independence allows us to define the score of a given tiling to be the product of the scores of the individual tiles it is composed of. The score of each individual tile is given by the data likelihood of that tile, as defined by Equation (8), multiplied by the prior probability of the tile's outbreak state. That is, the score of the hypothesis that tile *T *contains an outbreak is *p *⋅ *lik*(1, *T*) and the score of the hypothesis that it does not contain an outbreak is (1 - *p*) ⋅ *lik*(0, *T*).

To illustrate the effects of multiplying the likelihood by a prior, suppose that in the 1 × 2 example in Figure 2 the likelihoods of the tiles in Hypothesis 5 and Hypothesis 6 were the same, both equal to some value *l*. In that case the score of Hypothesis 6, which contains only one tile, would be *lp *while the score of Hypothesis 5, which contains two tiles, would have the lower value of *lp*^2^. Hence, all other things being equal, our prior favors tilings composed of fewer tiles.

A multiplicative prior for each tile is used to allow the decomposition of a tiling score into a product of individual tile scores. While we use the same prior for all tiles, it is possible to assign a different prior probability for each tile based on its location and size while maintaining the multiplicative property that the score of a tiling is a product of tile scored. Also note that while the priors for each tile add up to 1, the priors for tilings, which are a product of tile priors, are not normalized. We discuss the details of normalization when we present Bayesian model averaging, since it is especially relevant in that context. The process of selecting the value of the structure prior *p *and its relationship to the prior probability that an outbreak is present anywhere in the region (the "global prior") is also discussed alongside normalization. Below, let us denote the score of an outbreak state *x *for a tile *T *that spans rows *R_L _*through *R_H _*and columns *C_L _*through *C_H _*by

(26) $$score\left(x,R_{L},R_{H},C_{L},C_{H}\right)=score\left(x,T\right)=p^{x}{\left(1-p\right)}^{1-x}lik\left(x,T\right)$$

### Dynamic programming algorithm

It is clear that the space of possible outbreak hypotheses is exponential in the size of the grid, since there are 2^*R*×*C *^possible outbreak subregions and an even larger number of possible tilings. In order to efficiently search the space of hypotheses, a dynamic programming algorithm is presented for finding the most likely tiling that exploits the fact that the tiling of a large grid can be decomposed into multiple tilings of smaller grids.

**Figure 3:**
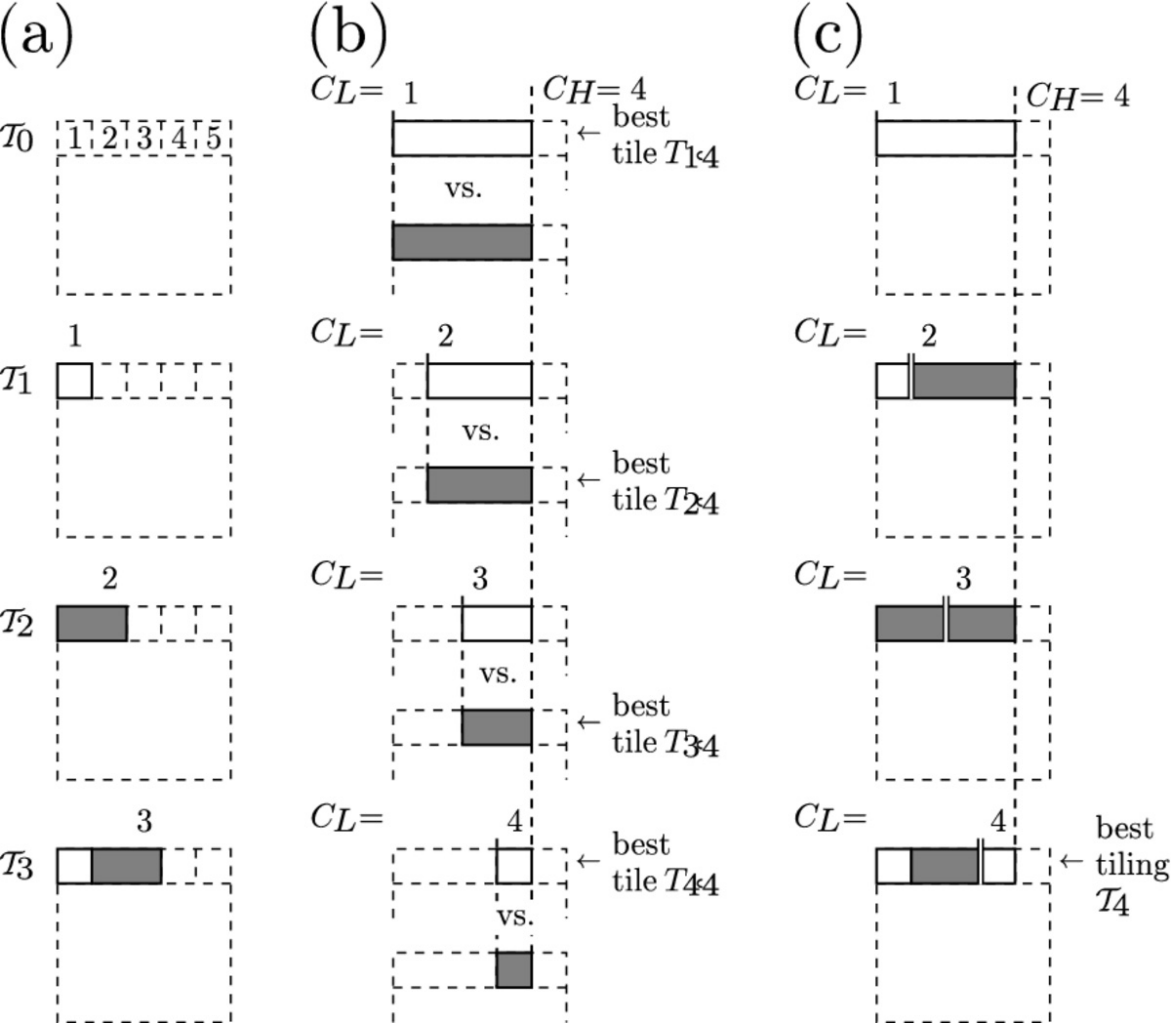
**One-dimensional tiling selection**. Illustration of an iteration of the tiling selection algorithm on a 1 × 5 strip of the grid. Tick marks indicate the boundaries of between the potential tile to be added and the rest of the tiling. See text for more details. (a) Best tilings $T_{C_{L}-1}$ for C_L _∈ {1, 2, 3, 4}. (b) Candidate tiles $T_{C_{L}C_{H}}$ for each value of C_L_. Highest scoring tile for each pair is shown as "best.". (c) Candidate tilings $T_{C_{L}-1}\cup \left\{T_{C_{L}C_{H}}\right\}$ for each value of C_L_. Highest scoring tiling shown as "best."

**Figure 4:**
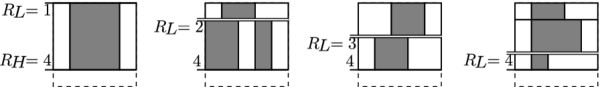
**Two-dimensional intermediate tilings**. Illustration of intermediate candidate tilings on a 5 × 5 grid for *R_H _*= 4.

To present the operation of the algorithm, we will first describe the algorithm for finding the highest-scoring tiling of a 1 × *C *horizontal strip in general terms, then walk through an example of tiling the top row of a 5 × 5 grid illustrated in Figure 3. Next we describe how the algorithm is extended to two dimensions with the aid of the example on a 5 × 5 grid illustrated in Figure 4, and finally we provide a formal definition of the algorithm as pseudocode.

In order to find the best (highest scoring) tiling of a 1 × *C *strip, we first number the cells consecutively from 1 to *C*. Let $T_{C_{L}-1}$ denote the best tiling of cells numbered *C_L _*- 1 (inclusive) and lower. When *C_L _*= 1, $T_{C_{L}-1}$ (or equivalently $T_{0}$) is a tiling of an empty set of cells. There is only one such possible tiling, the empty tiling, and we assign it a score of 1 because it is the multiplicative identity. Having laid out this grounding we can describe the operation of the algorithm iteratively: Given that we have found the best tiling $T_{C_{L}-1}$ for each *C_L _*such that 1 ≤ *C_L _*≤ *C_H_*, we can determine the best tiling $T_{C_{H}}$ for cells 1, ..., *C_H _*as follows: For each *C_L _*≤ *C_H_*, determine whether the tile $T_{C_{L}C_{H}}$ that spans the cells *C_L_*, ..., *C_H _*(inclusive) should be an outbreak tile or a non-outbreak tile to maximize its score, then multiply the score by the score of the best tiling $T_{C_{L}-1}$. This product is the score of the tiling obtained by appending tile $T_{C_{L}C_{H}}$ to tiling $T_{C_{L}-1}$, and this resulting tiling is then a candidate for tiling $T_{C_{H}}$. Then out of all the candidates obtained for the different values of *C_L _*in 1, ..., *C_H_*, the highest scoring one is guaranteed to be the highest scoring tiling of the range of cells 1, ..., *C_H_*. Thus we obtain the best tiling for the entire strip by iterating over values of *C_H _*from 1 to *C*.

Figure 3 illustrates a single iteration of this algorithm when looking for the best scoring tiling of the first (top) row of a 5 × 5 grid. The cells are numbered left to right. Particularly, the figure shows us the iteration that obtains the best tiling $T_{4}$ of the first (leftmost) four cells, thus, throughout this iteration, *C_H _*= 4. At this point, we have already obtained the best tilings of the lower ranges of cells $T_{0},\ldots ,T_{3}$ in previous iterations. These are shown in Figure 3(a). Figure 3(b) shows that for each value of *C_L _*from 1 through *C_H _*= 4, we consider whether an outbreak or a non-outbreak tile $T_{C_{L}C_{H}}$ scores higher. The best scoring tile from each pair is indicated. In Figure 3(c), we show each of those highest scoring tiles $T_{C_{L}C_{H}}$ combined with the corresponding best tiling $T_{C_{L}-1}$ from Figure 3(a). In our example suppose that of those resulting tilings the bottom one (obtained with *C_L _*= 4) resulted in the highest score. This tiling is then the best tiling $T_{4}$ of cells 1...4, and it is cached for use in the next iteration.

This method is extended to the two-dimensional case by performing the same iteration over rows, where each row takes the role analogous to a cell in the one-dimensional version discussed above. For example, Figure 4 is analogous to Figure 3(c) in that we know the best tiling for the rectangular region that spans rows 1 through *R_L _*- 1, and we are adding the column-wise tiling of the rectangular region spanning rows *R_L _*through *R_H _*to get a candidate tiling of the entire area above the line labeled *R_H_*. The main difference is that, where in Figure 3(b) we were considering whether to add an outbreak tile or a non-outbreak tile, in Figure 4 we are simply adding the best column-wise tiling of the range of rows between *R_L _*and *R_H_*, as is illustrated by the fact that the best tiling of row 1 comes from an extension of the example in Figure 3.

**Figure 5:**
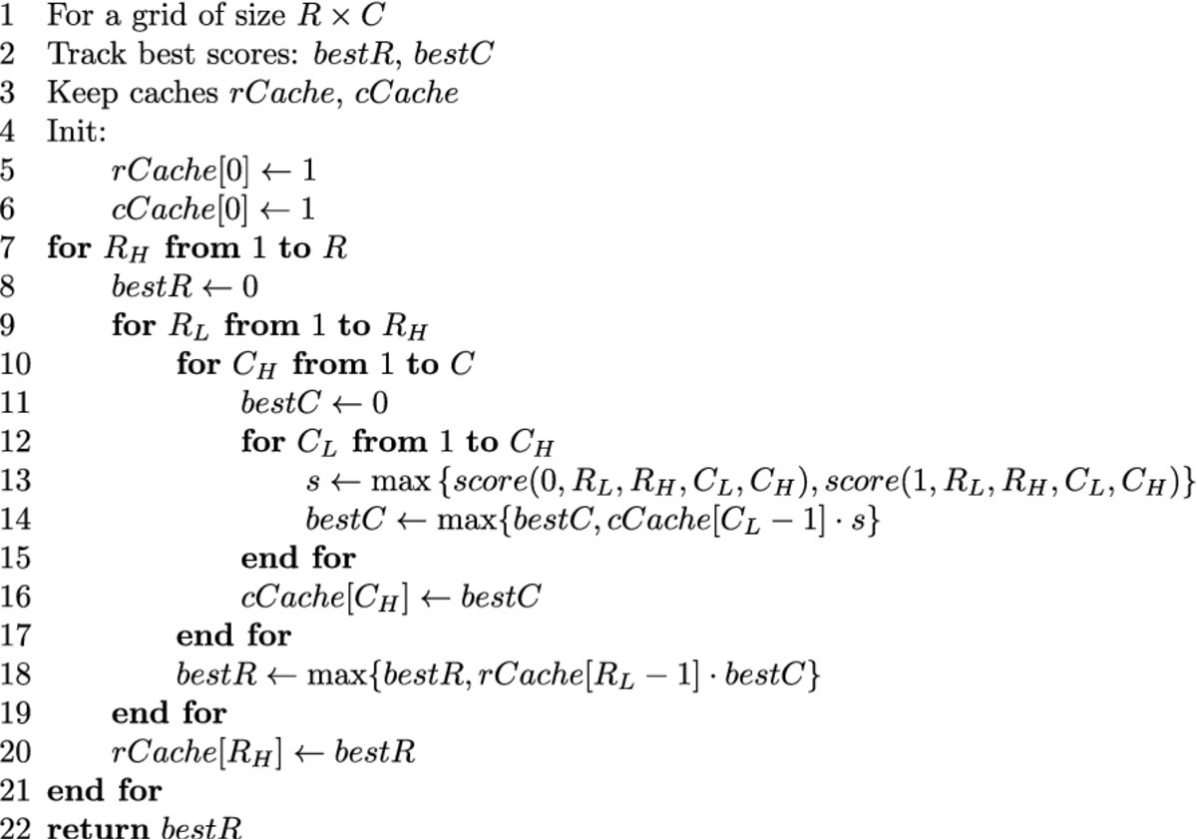
**Highest tiling score selection algorithm**. Pseudocode for the dynamic programming algorithm for highest tiling score selection.

For readability purposes, a version of the algorithm that only computes the score of the best tiling is presented in Figure 5. A version of the algorithm that keeps track of the actual tiling is detailed in Additional file 1: Appendix A.

The algorithm takes *Θ*(*R*^2 ^⋅ *C*^2^) iterations. In the general case, the computational cost of each iteration depends on the likelihood model used. In our particular implementation we compute a table of likelihoods for all possible grid rectangles prior to running the algorithm. Since our model is agent-based, the computational cost of populating the table is linear in the population of the surveillance region. The computational advantage that dynamic programming gives us is the ability to scan an exponential number of possible outbreak subregions. The exact number of possible tilings scanned is (2 × 3^*C*-1 ^+ 1)^*R*-1 ^× 2 × 3^*C*-1 ^= *Θ*(2^*R*(*Clg*3+1-*lg*3)^); of these tilings, (2^*C*-1 ^+ 1)^*R *-1 ^× 2^*C*-1 ^are non-outbreak hypotheses, and the rest are outbreak hypotheses, that is, hypotheses where at least one tile is colored. The calculation of the number of tilings is presented in Additional file 2: Appendix B.

We can express the value computed by the algorithm formally as follows: Let a row-wise partition of the grid be denoted by R∈Vwhere Vis the space of all row-wise partitions of the grid. Each partition R is a set of pairs of row indexes, where each pair (*R_L_*, *R_H_*) bounds the rectangular region *R_LH _*that spans the width of the grid (columns 1 through *C*) and rows *R_L _*through *R_H _*(inclusive). Let a column-wise partition of the rectangular region *R_LH _*be denoted by $C\in H\left(R_{L},R_{H}\right)$, where $H\left(R_{L},R_{H}\right)$ is the space of all column-wise partitions of *R_LH_*. We use the notation $\left(C_{L},C_{H}\right)\in C$ to mean that there is a tile spanning columns *C_L _*through *C_H _*(and rows *R_L _*through *R_H_*) in the partition. Using this notation, the algorithm finds:

(27) maxR∈V∏(RL,RH)∈R maxC∈H(RL,RH) ∏(CL,CH)∈C maxx∈{0,1}score(x,RL,RH,CL,CH)

**Figure 6:**
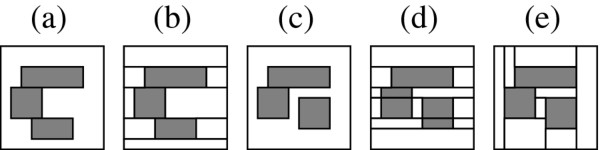
**Matching tilings to rectangles**. Two arrangements of rectangles and corresponding tilings that illustrate the capabilities and limitations of the dynamic programming algorithm's tilings. (a) The first sample outbreak configuration. (b) An optimal tiling of the first sample outbreak configuration that is within the algorithm's search space. (c) The second sample outbreak configuration. (d) The most optimal tiling of the second sample outbreak configuration within the algorithm's search space.(e)An optimal tiling of the second sample outbreak configuration outside the algorithm's search space.

The space of tilings considered by the algorithm can be described as the space of row-wise tilings of column-wise sub-tilings. Intuitively, this space has the desired property of being able to capture every cell-wise coloring of the grid, however, this is not an exhaustive search over all general colored tilings. Figure 6 illustrates this on a 10 × 10 grid with a pair of examples: Figures 6(a) and Figure 6(c) show two configuration of outbreak rectangles representing multiple outbreaks. It is desirable for a tiling found by the algorithm to be able to (1) cover all outbreak regions with outbreak tiles and all non-outbreak regions with non outbreak rectangles (that is, get the coloring right), (2) cover each outbreak rectangle with a single tile, and (3) minimize any unnecessary fragmentation of the non-outbreak region. For the outbreak configuration in 6(a), there exists a tiling in the search space of the algorithm that satisfy all three conditions, namely, the tiling in Figure 6(b). However, such a tiling does not always exist: The outbreak configuration in Figure 6(c) shows this limitation. A tiling in the space of all colored tilings that satisfies all three conditions is shown in Figure 6(e). Note that this tiling is not a row-wise tiling of column-wise sub-tilings and is hence not in the search space of the algorithm. In fact, a row-wise tiling that minimizes the fragmentation of the outbreak rectangles (to the extent possible for a row-wise tiling) is shown in Figure 6(d). Note that in Figure 6(d) the non-outbreak region has to be broken up into eleven tiles (as opposed to just eight in Figure 6(e)) and the three outbreak rectangles need to be broken up into five tiles. Thus, the most parsimonious row-wise tiling shown in Figure 6(d) is still not as parsimonious as the tiling in Figure 6(e).

### Model averaging

**Figure 7:**
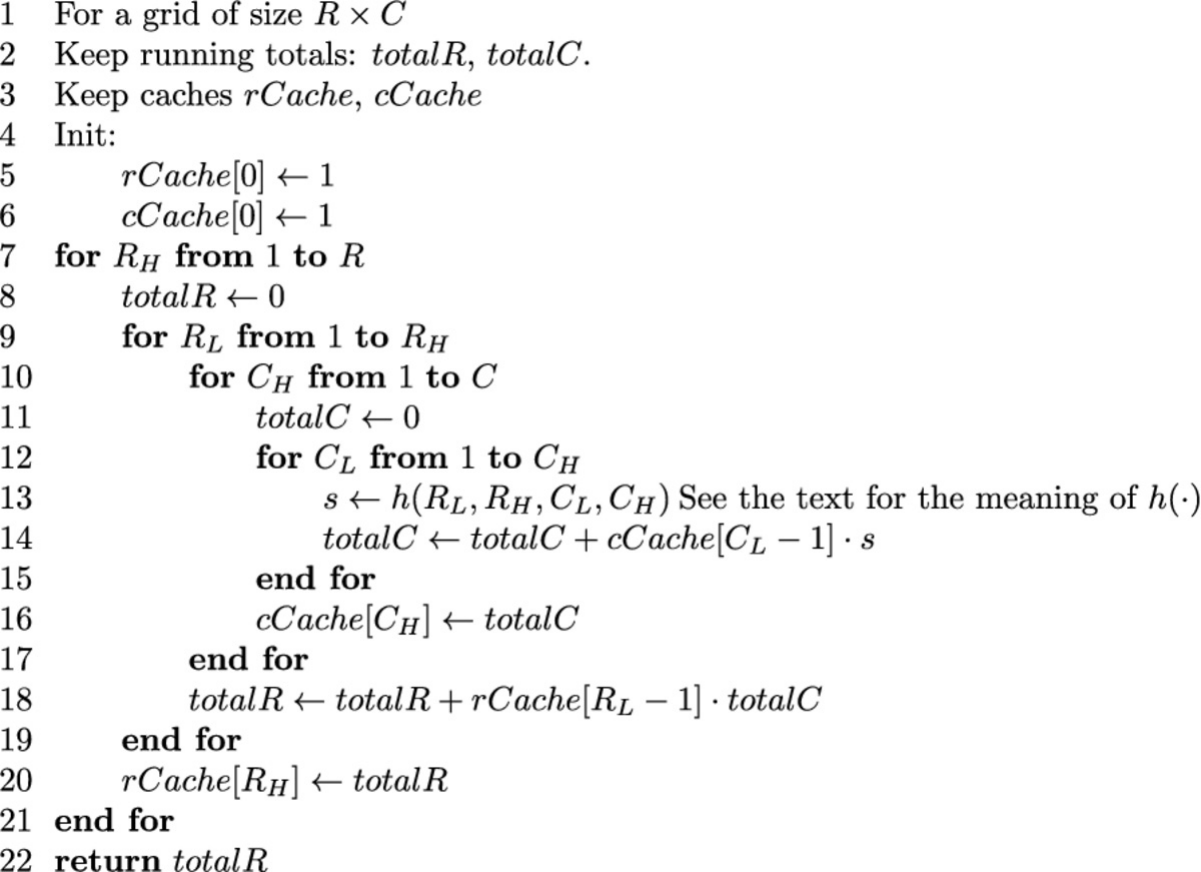
**Tiling summation algorithm**. Psuedocode for the dynamic programming algorithm for summing over tiling values that are products of individual tile values defined by a function *h*(⋅).

In the above algorithm it was assumed that the task at hand is model selection, that is, finding the most likely tiling given the data. Below we present an adaptation of the dynamic programming algorithm to perform Bayesian model averaging as well, in order to derive a posterior probability that an outbreak is occurring given the data. In simple terms, this is done by replacing maximization with summation to obtain two sums: *S*_0_(Data), the sum of the scores of all tilings that do not include outbreaks (blank tiles only); and *S*_01_(Data), the sum of the scores of all tilings considered. More specifically, replacing maximization by summation leads to the dynamic programming algorithm for summation over tilings in Figure 7.

The summation algorithm calculates the sum

(28) $$S_{h}=\underset{R\in V\;\;}{\sum }\;\underset{\left(R_{L},R_{H}\right)\in R\;\;}{\prod }\;\underset{C\in H\left(R_{L},R_{H}\right)\;\;}{\sum }\;\underset{\left(C_{L},C_{H}\right)\in C}{\prod }\;h\left(R_{L},R_{H},C_{L},C_{H}\right)$$

Here, the function *h*(*R_L_*, *R_H_*, *C_L_*, *C_H_*) defines the tile-wise values we would like to sum over. By substituting each of the following alternative functions for *h *we obtain the desired sums *S*_01_(*Data*) and *S*_0_(*Data*), respectively:

(29) $$h_{01}\left(R_{L},R_{H},C_{L},C_{H}\right)=score\left(0,R_{L},R_{H},C_{L},C_{H}\right)+score\left(1,R_{L},R_{H},C_{L},C_{H}\right)$$

(30) $$h_{0}\left(R_{L},R_{H},C_{L},C_{H}\right)=score\left(0,R_{L},R_{H},C_{L},C_{H}\right)$$

#### Score normalization

Under the above setup, the tiling scores need to be normalized in order to have the prior probabilities over all hypotheses sum to 1. The normalization constant *Z_T _*can be simply calculated as a sum over the priors associated with all tilings:

(31) $$\begin{array}{ccc} Z_{T} & =\underset{R\in V\;\;}{\sum }\;\underset{\left(R_{L},R_{H}\right)\in R\;\;}{\prod }\;\underset{C\in H\left(R_{L},R_{H}\right)\;\;}{\sum }\;\underset{\left(C_{L},C_{H}\right)\in C\;\;}{\prod }\;\underset{x\in \left\{0,1\right\}}{\sum }\;p^{x}{\left(1-p\right)}^{1-x} & \\ & =\underset{R\in V\;\;}{\sum }\;\underset{\left(R_{L},R_{H}\right)\in R\;\;}{\prod }\;\underset{C\in H\left(R_{L},R_{H}\right)\;\;}{\sum }\;\underset{\left(C_{L},C_{H}\right)\in C\;\;}{\prod }\;\left(\left(1-p\right)+p\right)\\ & =\underset{R\in V\;\;}{\sum }\;\underset{\left(R_{L},R_{H}\right)\in R\;\;}{\prod }\;\underset{C\in H\left(R_{L},R_{H}\right)\;\;}{\sum }\;\underset{\left(C_{L},C_{H}\right)\in C\;\;}{\prod }\;1 \end{array}$$

Hence the normalization constant *Z_T _*is simply the sum over tilings in (28) with *h *≡ 1. Additional file 3: Appendix C shows that for an *R *× *C *grid, this value can be calculated in closed form as *Z_T _*= *f*(*R*, *C*, *y*) using Equation (32) with *y *= 1:

(32) $$f\left(R,C,y\right)=y{\left(1+y\right)}^{C-1}{(1+y){(1+y)}^{C-1}}^{R-1}$$

Similarly, the prior probability, as governed by the structure prior parameter *p*, of the absence of an outbreak anywhere in the grid can be calculated as a sum over the priors of all non-outbreak tilings:

(33) $$\begin{array}{ccc} P\left(\text{no}\;\text{outbreak}\right) & =\frac{1}{Z_{T}}\underset{R\in V\;\;}{\sum }\;\underset{\left(R_{L},R_{H}\right)\in R\;\;}{\prod }\;\underset{C\in H\left(R_{L},R_{H}\right)\;\;}{\sum }\;\underset{\left(C_{L},C_{H}\right)\in C\;\;}{\prod }\;\left(1-p\right) & \\ & =\frac{f\left(R,C,1-p\right)}{Z_{T}}=\frac{f\left(R,C,1-p\right)}{f\left(R,C,1\right)} \end{array}$$

This relationship was used to pick the value of *p *that matches the particular value of *P(*outbreak) = 0.04 for the prior probability of an influenza outbreak based on expert assessment. Since we have not found a closed-form inverse to this relationship, the appropriate the value of *p *was found numerically using a binary search over numbers in [0,1].

In order to obtain the posterior probability of an outbreak, we normalize the sums of scores to obtain: *S*_0,1_(*Data*)/*Z_T _*= *P*(*Data*) and *S*_0_(*Data*)/*Z_T _*= *P*(Data, no outbreak). This allows us to obtain the posterior probability of an outbreak

(34) $$P\left(\text{outbreak}|\text{Data}\right)=1-P(\text{no}\;\text{outbreak}|\text{Data) = 1 - }\frac{P\left(\text{Data},\text{nooutbreak}\right)}{P\left(\text{Data}\right)}=1-\frac{S_{0}(\text{Data})}{S_{01}(\text{Data})}$$

In a typical biosurveillance application this is the posterior that we would use to detect whether an outbreak is occurring anywhere in the surveillance region by testing whether it rises above a pre-defined alert threshold.

### Evaluation methods

This section describes an evaluation of the dynamic programming (DP) algorithm, both in terms of model selection and in terms of model averaging. The evaluation was preformed using real background data consisting of information about chief complaints and home ZIP codes of ED patients who are presumed not to have an outbreak disease. Synthetic data were generated to simulate the presence of influenza outbreak cases. The evaluation consists of a comparison to a baseline that scans over single-rectangle hypotheses (SR), as well as a comparison for model selection against a greedy algorithm (GR) that hypothesizes one or more non-overlapping outbreak rectangles. The next section describes these algorithms and the section that follows it describes the data in further detail.

#### Baseline methods

The simpler of the baseline methods that the DP algorithm is compared to is a method of scanning over single-rectangle hypotheses (SR). For model selection, it consists of iterating over all possible placements of a single rectangle on the grid and finding the placement that maximizes the posterior probability of an outbreak, calculated as:

(35) $$P\left(H_{1}\left(S_{i}\right)|\text{Data}\right)=\frac{P\left(H_{1}\left(S_{i}\right)\right)\cdot lik\left(1,S_{i}\right)\cdot lik\left(0,{\bar{S}}_{i}\right)}{P\left(\text{Data}\right)}$$

where *S_i _*represents some rectangular region on the grid and ${\bar{S}}_{i}$ is its complement. In the implementation used in this evaluation, the prior probability *P*(*H*_1_(*S_i_*)) of having an outbreak in rectangle *S_i _*is set to be equal for all rectangles, hence maximization of the posterior probability here is equivalent to maximization of the likelihood $lik\left(1,S_{i}\right)\cdot lik\left(0,{\bar{S}}_{i}\right)$. The prior probabilities *P*(*H*_1_(*S_i_*)) are chosen so that the prior probability *P(*Outbreak) of an outbreak anywhere in the region matches the one for the dynamic programming algorithm.

Let the notation *S*(*R_L_*, *R_H_*, *C_L_*, *C_H_*) denote the rectangle *S_i _*defined by those row and column boundaries, and let $P\left(\text{Data},H_{1}\left(S_{i}\right)\right)=P\left(H_{1}\left(S_{i}\right)\right)\cdot lik\left(1,S_{i}\right)\cdot lik\left(0,{\bar{S}}_{i}\right)$. Then model averaging using the SR algorithm consists of simply calculating the posterior:

(36) $$P\left(\text{Outbreak}|\text{Data}\right)=\frac{\overset{R}{\underset{R_{H}=1}{\sum }}\;\overset{R_{H}}{\underset{R_{L}=1}{\sum }}\;\overset{C}{\underset{C_{H}=1}{\sum }}\;\overset{C_{H}}{\underset{C_{L}=1}{\sum }}\;P\left(\text{Data},H_{1}\left(R_{L},R_{H},C_{L},C_{H}\right)\right)}{P\left(H_{0}\right)\cdot lik\left(0,S\left(1,R,1,C\right)\right)+\overset{R}{\underset{R_{H}=1}{\sum }}\;\overset{R_{H}}{\underset{R_{L}=1}{\sum }}\;\overset{C}{\underset{C_{H}=1}{\sum }}\;\overset{C_{H}}{\underset{C_{L}=1}{\sum }}\;P\left(\text{Data},H_{1}\left(R_{L},R_{H},C_{L},C_{H}\right)\right)}$$

The other baseline method that is used in the evaluation is the greedy algorithm (GR) without recursion by Jiang and Cooper [18]. We use this algorithm for model selection only, and it operates iteratively: First it selects the single rectangle $S_{i_{1}}$ that maximizes the score $q\cdot lik\left(1,S_{i_{1}}\right)\cdot lik\left(0,{\bar{S}}_{i_{1}}\right)$, where *q *is a structure prior. Next the non-overlapping rectangle $S_{i_{2}}$ that maximizes the score $q\cdot lik(1,S_{i_{1}})\cdot q\cdot lik(1,S_{i_{2}})\cdot lik(0,\bar{S_{i_{1}}\cup S_{i_{2}}})$ is selected. This process of adding one rectangle at each step and multiplying by the structure prior *q *with each new added rectangle is repeated until the score can no longer be increased. The result is a collection of non-overlapping rectangles $\left\{S_{i_{1}},\ldots ,S_{i_{n}}\right\}$ with the associated score

(37) $$q^{n}\cdot lik\left(0,\bar{\overset{n}{\underset{j=1}{\cup }}\;S_{i_{j}}}\right)\cdot \prod_{j=1}^{n}\;lik(1,S_{i_{j}})$$

Outbreak rectangles are considered to be independent conditioned on their positions. The value of *q *is chosen to be equal to the value used in the SR algorithm for *P*(*H*_1_(*S_i_*)).

### Data generation

Multiple outbreak scenarios were generated on a 10 × 10 grid with a population distribution based on ZIP Code Tabulation Area populations in Allegheny County, Pennsylvania as reported in the 2000 Census. Real ED data with home ZIP code information was used as a non-outbreak background scenario. Data from the months of June-August of the years 2003-2005, a total of 181 days, were used to evaluate the background scenario. The data for each day consisted of a de-identified list of records for patients who visited emergency departments in Allegheny county on that day, where each record contained the patient's home zip code and chief complaints. Ethics approval for use of the data was provided by the University of Pittsburgh IRB.

Outbreaks of varying shapes, sizes, and frequencies were then injected into the data to obtain outbreak scenarios. In simulating outbreaks, the outbreak frequency *F *was sampled as a continuous uniform random variable from one of the following four frequency ranges: low (0 to 5.91 × 10^-5^), mid (5.91 × 10^-5 ^to 3.55 × 10^-4^), high (3.55 × 10^-4 ^to 6.50 × 10^-4^), and very high (6.50 × 10^-4 ^to 0.5).

**Figure 8:**
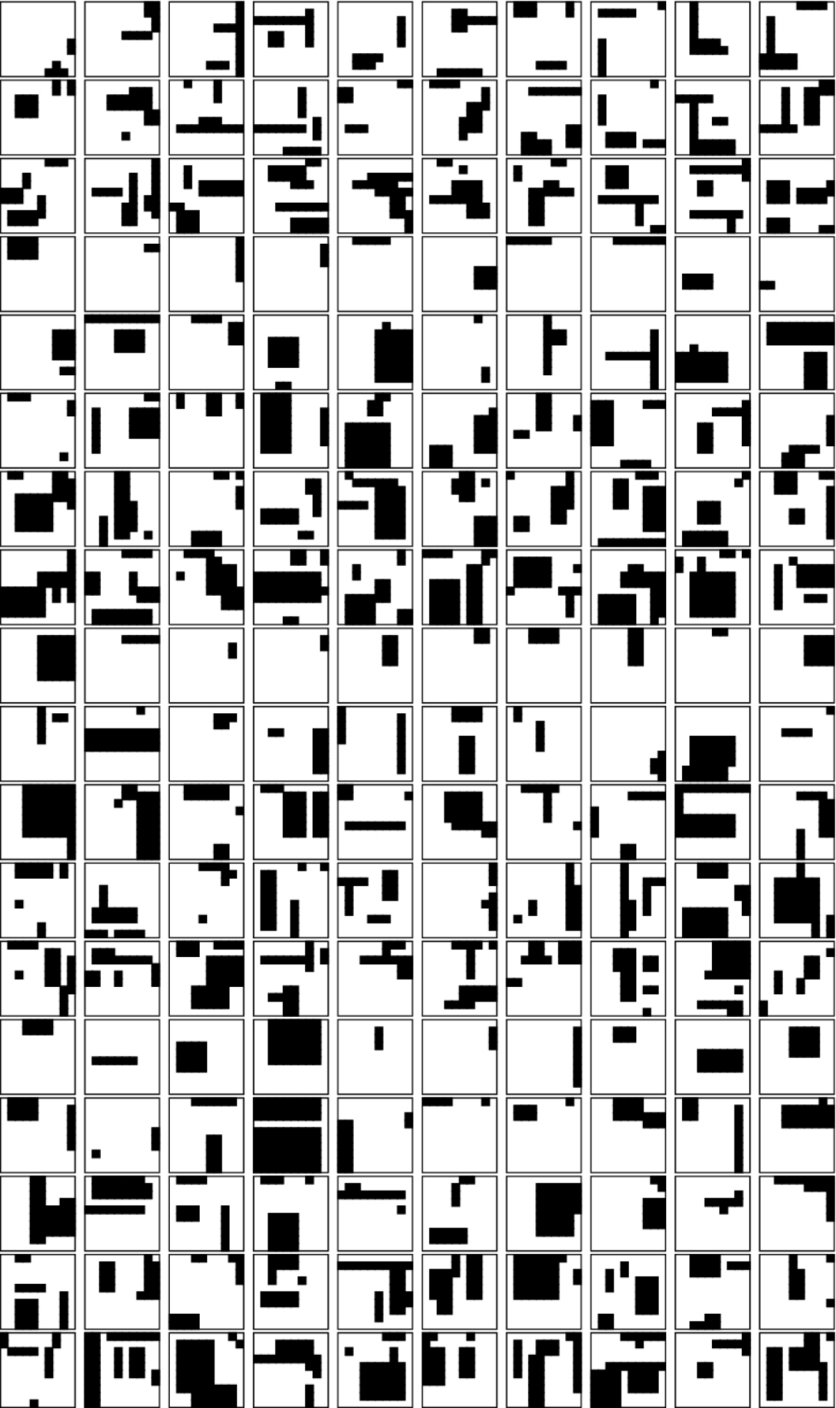
**Randomly generated outbreaks**. A sample of some randomly generated outbreak shapes for testing.

For each frequency range, four sets of outbreak specifications were generated where the rectangle sizes were limited to a maximum of 10, 40, 60, and 100 cells. Each of these sets was in turn composed of five sets of specifications with *n *= 1 to 5 non-overlapping rectangles, 10 scenarios for each value of *n*, giving a total of 800 outbreak specifications. The positioning of each rectangle on the grid was uniformly random. Figure 8 shows a sample of the randomly generated outbreak shapes that were used. Each rectangle *j *was assigned an outbreak frequency *F_j _*sampled from the previously determined frequency range.

For each of the 181 days of background data, each of the 800 outbreak specifications was used to create an outbreak scenario by injecting disease cases into that day by selecting an individual in a an outbreak rectangle indexed by *j *with probability *F_j _*and setting the corresponding symptom *I_i _*by sampling the distribution of *P*(*I_i_*|*D_i _*= *flu*) defined in our disease model.

For each outbreak scenario, the outbreak's coverage (the proportion of the grid that is covered by outbreak rectangles) was also recorded. This value is later used to stratify the test results.

## Results and discussion

### Evaluation of outbreak presence detection

We evaluated model averaging of the dynamic programming (DP) and the single rectangle (SR) algorithms by obtaining the posteriors for each day in the background data (when no outbreak is occurring) and for each day in the outbreak scenarios, and aggregating these results to obtain Receiver Operating Characteristic (ROC) curves. Note that days are treated as single snapshots of the surveillance region with no temporal context, and the area under the ROC curve gives us a measure of the quality of detection of an outbreak from observing only a single day of the outbreak regardless of how far into the outbreak's progression that day is. This is unlike another popular metric in the outbreak detection literature, the Activity Monitoring Operating Characteristic (AMOC) curve, where a progressing outbreak is simulated over a span of multiple days and the time to detection is measured. We use an evaluation measure that does not take time into account because the algorithms in question themselves are purely spatial. Additionally, the need to simulate outbreak progression for AMOC analysis inadvertently introduces additional assumptions about the dynamics of the outbreaks to be detected, which is not the focus of the current evaluation.

In these tests the disease model used assumes a single-valued *K*. Table 1 reports the areas under the curve (AUC) and the results of a statistical significance test based on [25] of the difference in areas under the curve of the two methods. The table shows the AUC for each algorithm, the lower and upper 95% confidence limits for the difference in areas (DP-SR) and the associated *p*-values. Note that in the mid-range frequencies and above, the posteriors for all outbreaks with coverage 35% and above were all equal to 1 up to numerical precision for both methods. As a result, the ROC curves obtained for those samples are identical making the comparison trivial. In the "very high" frequency range all outbreaks yielded posteriors that are 1 up to numerical precision, and for this reason this frequency range is omitted from Table 1.

**Table 1:** Model averaging comparison in terms of area under the ROC curve

Frequency range	Coverage	DP AUC	SR AUC	(DP-SR) 95% confidence interval limits		*p*-value
Low	< 16%	0.95047	0.95016	- 0.00746	0.00808	0.9384
	
	16-34%	0.86260	0.86852	- 0.01261	0.00078	0.0831
	
	≥ 35%	0.97409	0.97911	- 0.01005	0.00001	0.0505

Mid	< 16%	0.96019	0.96079	- 0.00530	0.00410	0.8014
	
	16-34%	0.98246	0.98613	- 0.00575	- 0.00159	0.0005
	
	≥ 35%	1.00000	1.00000			

High	< 16%	0.98468	0.98379	- 0.00173	0.00352	0.5044
	
	16-34%	0.99990	0.99984	- 0.00001	0.00013	0.1096
	
	≥ 35%	1.00000	1.00000			

Comparison of the DP and SR ROC curves obtained from model averaging. The table shows the AUC for each algorithm, the lower and upper 95% confidence limits for the difference in areas (DP-SR) and the associated p-values

The results show that both methods perform similarly and do not achieve statistically different results, except in the case of mid-range frequencies with outbreak coverage of 16-34% of the surveillance grid where the SR method performs statistically significantly better, but the performance difference was inconsequential from a practical standpoint. We can also see that for both methods the area under the ROC curve is strongly influenced by both the frequency and coverage of the outbreak, increasing as these parameters increase, as expected.

#### Additional investigation

The result that the DP algorithm does not perform better than the SR algorithm is surprising since the DP algorithm is able to consider multiple-rectangle hypotheses which would be expected to drive the posterior probability up significantly when there are multiple simulated outbreaks.

**Figure 9:**
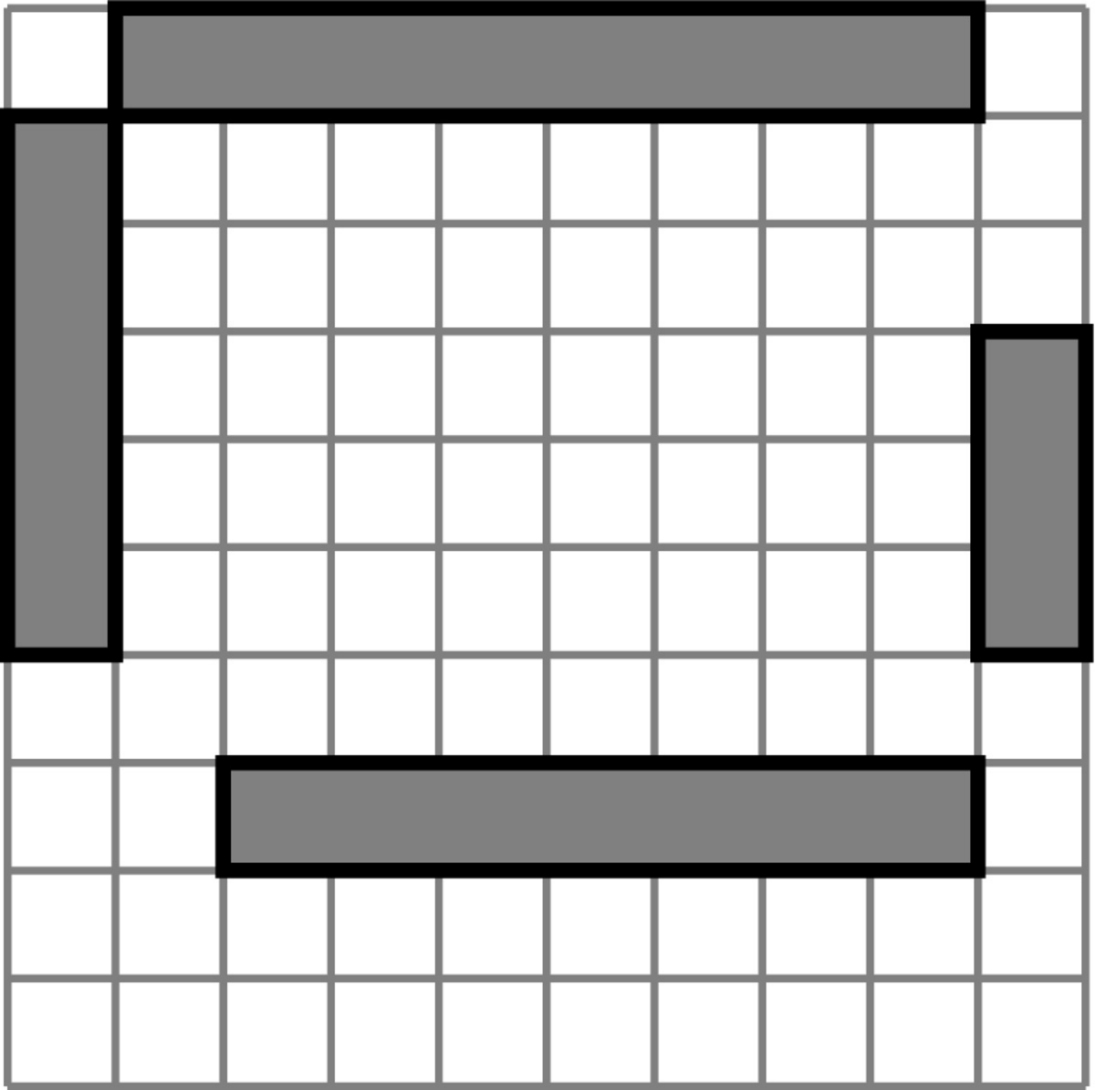
**Doughnut-shaped outbreak**. An example of a specially designed outbreak for further comparison of SR and DP model averaging.

Since it is not immediately obvious from inspection of the algorithms why the anticipated improvement is absent, we performed targeted tests with the same background data and specially-designed outbreaks, such as the one illustrated in Figure 9. Such "doughnut shaped" outbreaks are expected to be more challenging for the SR algorithm because any placement of a single rectangle on the grid will either miss a significant portion of the outbreak cells or include a significant number of non-outbreak cells. The outbreak shape in Figure 9 and the possible combinations of three, two, and one rectangle of the four shown in Figure 9 (making a total of 15 different outbreak shapes) were injected into the background data at the low, mid, and high frequency ranges to obtain ROC curves. As the statistical comparison in Table 2 shows, these tests show that even in multi-rectangle outbreaks designed to be difficult to approximate with a single-rectangle cluster, the SR algorithm performs at least as well as the DP algorithm in terms of area under the ROC curve.

**Table 2:** Additional model averaging comparison

Frequency range	Number of rectangles	DP AUC	SR AUC	**(DP-SR) 95% confidenceinterval limits**		*p*-value
Low	1	0.51545	0.51647	- 0.01450	0.01247	0.8830
	
	2	0.52214	0.52353	- 0.01462	0.01185	0.8373
	
	3	0.55150	0.55355	- 0.01652	0.01244	0.7824
	
	4	0.55416	0.55637	- 0.02003	0.01561	0.8080

Mid	1	0.66873	0.67618	- 0.01967	0.00478	0.2326
	
	2	0.80826	0.82422	- 0.02771	- 0.00420	0.0078
	
	3	0.78402	0.79984	- 0.03444	0.00650	0.0520
	
	4	0.88198	0.88272	- 0.01471	0.01324	0.9177

High	1	0.88433	0.91615	- 0.04173	- 0.02191	< 0.0001
	
	2	0.96696	0.97218	- 0.01147	0.00105	0.1027
	
	3	0.97819	0.98139	- 0.00749	0.00109	0.1438
	
	4	0.99626	0.99567	- 0.00224	0.00340	0.6852

Comparison DP and SR ROC curves obtained from model averaging for outbreaks composed of various combinations of the rectangles in Figure 9

In light of these results we also followed another line of investigation, namely, one of changing the underlying likelihood function with the thought that a model that more accurately represents the background scenarios would yield more representative likelihoods, and possibly better differentiation between the nuances of the two algorithms. Since in reality the proportion *K *of people coming to the ED for non-outbreak diseases varies from day to day, modeling *K *as a random variable with a nontrivial distribution is more realistic. Under this new model, the DP algorithm performed similarly to its performance under the old model. The SR algorithm, however, took a severe turn for the worse as it marked almost every non-outbreak scenario with a very high posterior probability, numerically indistinguishable from one. For this reason, we do not present a comparison between the methods using the distribution-based model. We have carefully checked the correctness of the implementation of the SR algorithm and must therefore conclude that this behavior is indeed a drawback of the the SR algorithm. Further investigation is required to fully understand this behavior, however.

#### Comparison to SaTScan™

We have compared DP to SaTScan™,^a ^the software package developed by Kulldorff that can use spatial, temporal, or space-time scan statistics [26]. SaTScan™ implements a popular set of algorithms that (1) have been extensively evaluated by its creators, (2) perform well in practice, (3) are made available for free download for research purposes, and (4) have been used by others as a benchmark ofscanning performance. Therefore, we believe SaTScan™ provides a good point of comparison to DP, although we describe below some caveats regarding this comparison. Since the current implementation of DP is purely spatial, we restricted the comparison only to the spatial scan statistic.

It is important to note that there are fundamental differences between the DP algorithm and SaTScan™. Firstly, SaTScan™ is designed to detect clusters of higher or lower than normal values of some measurement and report those as clusters, while our method uses a disease model to calculate a likelihood of a state of interest based on observations. This means that in our testing, we cannot use SaTScan™ to directly detect high numbers of influenza cases *per se*, since the disease state is never directly observed in the data. For this reason, we used SaTScan™ to detect abnormally high counts of patients that came in with a chief a complaint of either "cough" or "fever", as they are indicative of the locations of influenza cases. We leave the information provided to the DP algorithm unchanged (the values of the chief complaint variable are provided) in this comparison. Secondly, SaTScan™ does not scan over rectangular regions, but rather over circular or ellipse-shaped regions [4]. We still use the same generated outbreaks for this comparison, meaning that the true outbreaks are rectangular. SaTScan™ does have the ability to detect multiple clusters in an iterative process that is similar to the greedy algorithm. In this mode, SaTScan™ scans for the most likely ellipse-shaped cluster, then removes the data it captures, and repeats the process until the *p*-value (the probability of the observed data under the null hypothesis that no cluster exists) of the newly detected cluster falls above 0.05 [27]. Since this is the setting which is most suited for detecting multiple and irregularly-shaped clusters, it is the one we used in our comparison. Of the models available to SaTScan™ we used the Poisson-based model [3] since it is the most appropriate for the setting where we are detecting abnormally high event counts for a known population at risk.

Table 3 reports the AUC and the statistical significance of ROC differences comparing the DP algorithm to SaTScan™. In order to obtain ROC curves from SaTScan™, we used the *p*-value of the most likely cluster found as the criterion variable, with lower *p*-values corresponding to a positive decision (in favor of an outbreak). The table shows that SaTScan™ performs statistically significantly worse than DP in all tests.

**Table 3:** DP compared to SaTScan™ in terms of area under the ROC curve

Frequency range	Coverage	DP AUC	SaTScan™	(DP-SaTScan™) 95% confidence interval limits		*p*-value
Low	< 16%	0.95047	0.49570	0.41018	0.49936	< 0.0001
	
	16-34%	0.86260	0.46070	0.35816	0.44562	< 0.0001
	
	≥ 35%	0.97409	0.42947	0.50375	0.58549	< 0.0001

Mid	< 16%	0.96019	0.65946	0.26789	0.33356	< 0.0001
	
	16-34%	0.98246	0.85287	0.11099	0.14818	< 0.0001
	
	≥ 35%	1.00000	0.81147			

High	< 16%	0.98468	0.82496	0.14089	0.17856	< 0.0001
	
	16-34%	0.99990	0.99490	0.00387	0.00611	< 0.0001
	
	≥ 35%	1.00000	0.94950			

Comparison of the DP posteriors obtained from model averaging and SaTScan™ p-values in terms of ROC curves. The table shows the AUC for each algorithm, the lower and upper 95% confidence limits for the difference in areas (DP-SaTScan™) and the associated p-values

A possible factor influencing the poor performance of SaTScan™ is that the task that it performs is not a perfect fit for our evaluation measure. We are measuring performance in the task of identifying injected disease outbreaks, while SaTScan™ performs the task of detecting elevated counts of the "cough" or "fever" chief complaint. Also, we injected rectangular outbreaks, whereas SaTScan™ is looking for elliptical shaped outbreak regions. Another potential factor to the performance of SaTScan™ is that the background (no-outbreak) scenarios also contain clusters of elevated chief complaint counts of cough and fever, even in the absence of injected influenza outbreak cases. DP itself is not free from the same problem, however, since it does assign high posteriors to some background scenarios, but to a lower extent.

### Evaluation of outbreak location detection

In order to compare the quality of the detection of the location of outbreak clusters, the cell-wise spatial precision and recall of each algorithm were measured in each outbreak scenario. In terms of the most likely outbreak subregion *Q *detected by each algorithm and the actual subregion SUB covered by the simulation-generated outbreak rectangles in each scenario, the cell-wise spatial precision is taken to be the proportion |*Q *∩ *SUB*|/|*Q*| and the cell-wise spatial recall to be |*Q *∩ *SUB*|/|*SUB*| where the notation |*X*| signifies the area of subregion *X*. The measurement is restricted to populated cells only.

The disease model used in these tests is also the one that assumes a single-valued *K*, due to details of the greedy algorithm implementation. Recall from equation (37) that the likelihood of an irregular region, $lik\left(0,\bar{\cup_{j=0}^{n}\;S_{i_{j}}}\right)$ needs to be calculated at each step. When *K *is a distribution, this calculation needs to be re-done for every considered hypothesis because the expression is a sum of products, leading to much longer runtime. However, when *K *is a single value, since the likelihood of that region is a simple product, we can take advantage of canceling terms and calculate it much faster from cached values as

(38) $$lik\left(0,\bar{\overset{n}{\underset{j=0}{\cup }}\;S_{i_{j}}}\right)=\frac{lik(0,1,R,1,C)}{\prod_{j=0}^{n}\;lik(0,S_{i_{j}})}$$

Table 4 shows the average cell-wise spatial precision for the DP, GR, and SR methods. The table also shows the result of paired *t*-tests for significance of the difference between the DP and GR algorithms. Since it is clear that the single-rectangle algorithm performs worse than the other two, the statistical comparison to SR is not shown here. Table 5 shows a similar table for cell-wise recall.

**Table 4:** Comparison of model selection in terms of spatial precision

Frequency range	Coverage	DP precision	GR precision	SR precision	(DP-GR) 95% confidence interval limits		*p*-value
Low	< 16%	0.09365	0.11775	0.07022	- 0.02412	- 0.02408	< 0.00001
	
	16-34%	0.28551	0.33207	0.27251	- 0.04659	- 0.04654	< 0.00001
	
	≥ 35%	0.57974	0.62428	0.60255	- 0.04462	- 0.04447	< 0.00001

Mid	< 16%	0.31987	0.34414	0.11212	- 0.02430	- 0.02425	< 0.00001
	
	16-34%	0.67969	0.66288	0.31934	0.01679	0.01683	< 0.00001
	
	≥ 35%	0.92269	0.86046	0.55602	0.06211	0.06235	< 0.00001

High	< 16%	0.50417	0.53018	0.15127	- 0.02604	- 0.02599	< 0.00001
	
	16-34%	0.84815	0.78839	0.34174	0.05974	0.05978	< 0.00001
	
	≥ 35%	0.91292	0.83541	0.54478	0.07740	0.07763	< 0.00001

Very high	< 16%	0.66221	0.51208	0.14081	0.15010	0.15017	< 0.00001
	
	16-34%	0.84584	0.44728	0.26127	0.39854	0.39859	< 0.00001
	
	≥ 35%	0.92306	0.66563	0.48019	0.25728	0.25758	< 0.00001

Comparison of the DP, GR, and SR spatial precisions obtained from model selection. The table shows the mean precision for each algorithm, the lower and upper 95% confidence limits for the difference in precision (DP-GR) and the associated *t*-test p-values

**Table 5:** Comparison of model selection in terms of spatial recall

Frequency range	Coverage	DP recall	GR recall	SR recall	**(DP-GR) 95% confidence interval limits**		*p*-value
Low	< 16%	0.08729	0.09759	0.04993	- 0.01031	- 0.01029	< 0.00001
	
	16-34%	0.13665	0.14801	0.10795	- 0.01137	- 0.01135	< 0.00001
	
	≥ 35%	0.18579	0.19636	0.17592	- 0.01060	- 0.01055	< 0.00001

Mid	< 16%	0.39280	0.38533	0.11793	0.00745	0.00749	< 0.00001
	
	16-34%	0.56118	0.55025	0.24091	0.01092	0.01095	< 0.00001
	
	≥ 35%	0.74869	0.75813	0.42978	- 0.00956	- 0.00932	< 0.00001

High	< 16%	0.62454	0.60666	0.13601	0.01786	0.01789	< 0.00001
	
	16-34%	0.82999	0.82147	0.29433	0.00850	0.00852	< 0.00001
	
	≥ 35%	0.91850	0.91130	0.50550	0.00716	0.00724	< 0.00001

Very high	< 16%	0.93248	0.92970	0.24123	0.00277	0.00278	< 0.00001
	
	16-34%	0.99829	0.99809	0.62275	0.00020	0.00020	< 0.00001
	
	≥ 35%	0.99813	0.99815	0.74743	- 0.00002	- 0.00001	< 0.00001

Comparison of the DP, GR, and SR spatial recall obtained from model selection. The table shows the mean recall for each algorithm, the lower and upper 95% confidence limits for the difference in recall (DP-GR) and the associated *t*-test p-values

First note that all differences observed between the DP and GR algorithms, while small, are statistically significant at the large sample sizes used. In terms of both spatial precision and recall, the results show that the greedy algorithm does slightly better than the dynamic programming algorithm at low frequencies, even though the performance of both is very poor. This may be an indication that the greedy algorithm is more sensitive to small differences in likelihood. For the most part, as frequency and coverage of the outbreak increase, the performance of the dynamic programming algorithm overtakes that of the greedy algorithm. Most notably, the dynamic programming algorithm yields much better spatial precision for outbreaks with higher spatial coverage (and therefore more outbreak rectangles). The differences in recall, while statistically significant, do not amount to much from a practical standpoint.

We also compare the DP algorithm to SaTScan™ in terms of spatial precision and recall. Table 6 compares the algorithms in terms of spatial precision and Table 7 compares the algorithms in terms of spatial recall. In order to obtain precision and recall readings from SaTScan™, we considered locations that are contained in a detected cluster with a *p*-value below 0.05 to be marked as outbreak locations, and all others to be marked as non-outbreak locations.

**Table 6:** Comparison of spatial precision against SaTScan™

**Frequencyrange**	Coverage	DP precision	SaTScan™ precision	**(DP-SaTScan™) 95%confidence interval limits**		*p*-value
Low	< 16%	0.09365	0.09218	0.00395	- 0.00100	0.12159
	
	16-34%	0.28551	0.27073	0.01871	0.01084	< 0.00001
	
	≥ 35%	0.57974	0.47264	0.11638	0.09781	0.00128

Mid	< 16%	0.31987	0.24489	0.07877	0.07118	< 0.00001
	
	16-34%	0.67969	0.59986	0.08355	0.07612	< 0.00001
	
	≥ 35%	0.92269	0.87176	0.05817	0.04369	< 0.00001

High	< 16%	0.50417	0.33449	0.17408	0.16526	< 0.00001
	
	16-34%	0.84815	0.66405	0.18728	0.18093	< 0.00001
	
	≥ 35%	0.91292	0.82920	0.08906	0.07839	< 0.00001

Very high	< 16%	0.66221	0.41769	0.24879	0.24027	< 0.00001
	
	16-34%	0.84584	0.62706	0.22190	0.21568	< 0.00001
	
	≥ 35%	0.92306	0.79866	0.11971	0.12911	< 0.00001

Comparison of the DP and SaTScan™ spatial precision. The table shows the mean recall for each algorithm, the lower and upper 95% confidence limits for the difference in recall (DP-SaTScan™) and the associated *t*-test p-values

**Table 7:** Comparison of spatial recall against SaTScan™

Frequency range	Coverage	DP recall	SaTScan™ recall	(DP-SaTScan™) 95% confidence interval limits		*p*-value
Low	< 16%	0.08729	0.15045	- 0.05982	- 0.06649	< 0.00001
	
	16-34%	0.13665	0.18430	- 0.04485	- 0.05045	< 0.00001
	
	≥ 35%	0.18579	0.16384	0.02682	0.01708	< 0.00001

Mid	< 16%	0.39280	0.37422	0.02227	0.01489	< 0.00001
	
	16-34%	0.56118	0.48644	0.07769	0.07180	< 0.00001
	
	≥ 35%	0.74869	0.58639	0.17157	0.15305	< 0.00001

High	< 16%	0.62454	0.49413	0.13376	0.12706	< 0.00001
	
	16-34%	0.82999	0.70011	0.13170	0.12805	< 0.00001
	
	≥ 35%	0.91850	0.76347	0.15955	0.15052	< 0.00001

Very high	< 16%	0.93248	0.69904	0.23695	0.22993	< 0.00001
	
	16-34%	0.99829	0.78494	0.21539	0.21132	< 0.00001
	
	≥ 35%	0.99813	0.78448	0.21034	0.21697	< 0.00001

Comparison of the DP and SaTScan™ spatial recall. The table shows the mean recall for each algorithm, the lower and upper 95% confidence limits for the difference in recall (DP-SaTScan™) and the associated *t*-test p-values

The spatial precision results show that SaTScan™ achieves precision not statistically significantly different from DP for outbreaks of low coverage and frequency, while for other cases DP achieves statistically significantly higher precision. The spatial recall results show that SaTScan™ achieves recall that is statistically significantly higher than DP for outbreaks of coverage below 35% and low frequency. Both methods perform generally poorly in this range. In the remainder of the cases DP outperforms SaTScan™ in terms of recall as well as precision.

We emphasize that DP has the advantage of performing inference about whether an individual has influenza using the same disease model that was used to generate the injected outbreaks. SaTScan™, however, merely searches for elevated chief complaint counts. Also note that the injected outbreaks are rectangular in shape, while SaTScan™ uses elliptical search windows. An ellipse is not able to fit some of the outbreak scenarios well. Additionally, unlike our model which has prior parameters defining a distribution over the chief complaints that are observed when no outbreak is present, SaTScan™ computes the expected counts for the absence of a cluster based on the counts outside the search window. As a result, when an elliptical window fails to accurately enclose the outbreak, SaTScan™'s assessment of the expected level of chief complaint counts is affected. We believe that the combination of these factors is what is responsible for SaTScan™'s lower performance.

### Evaluation of running time

A major motivating factor for developing a dynamic programming spatial scan is that brute force multi-region spatial scans are computationally intensive. In order to evaluate the feasibility of using the DP algorithm in a practical application setting, we performed a variety of timing tests on input data of varying grid sizes and populations. The population size affects the running time not due to any feature of the dynamic programming itself, but rather due to the agent-based disease model. In order to properly interpret the timing results, it is first useful to briefly describe some technical aspects of our implementation.

The implementation consists of two modules, one module reads in the data to be scanned and uses the disease model to compute the log-likelihood of each possible grid rectangle for each value of the flu incidence frequency *F *(and the "other" disease state frequency *K *when it is a distribution rather than a constant), including *F *= 0, and stores this table of likelihoods in a data structure. The second module is the actual DP scanning algorithm which queries the data structure constructed by the first module every time the likelihood of a tile needs to be calculated. We actually reuse the first module in the implementations of the SR and greedy algorithms since queries to the same tables of likelihoods need to be made by each of those algorithms as well.

**Figure 10:**
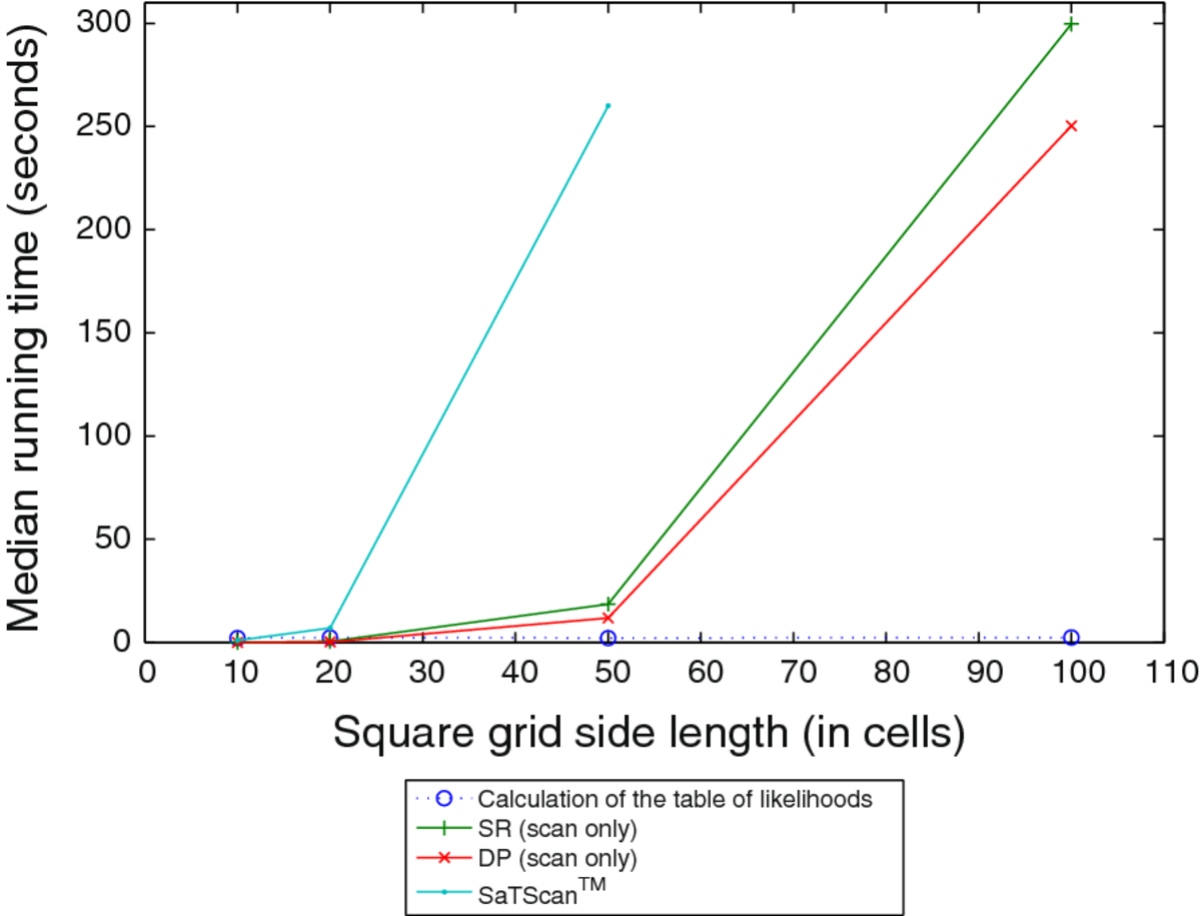
**Comparison of running time across a range of grid sizes**. Comparison of running time across grid sizes of 10 × 10, 20 × 20, 50 × 50, and 100 × 100 using the original 2000 census population of Allegheny County. The plot shows the running time of the calculation of the table of likelihoods, the scan of the SR algorithm, the scan of the DP algorithm, and SaTScan™.

**Figure 11:**
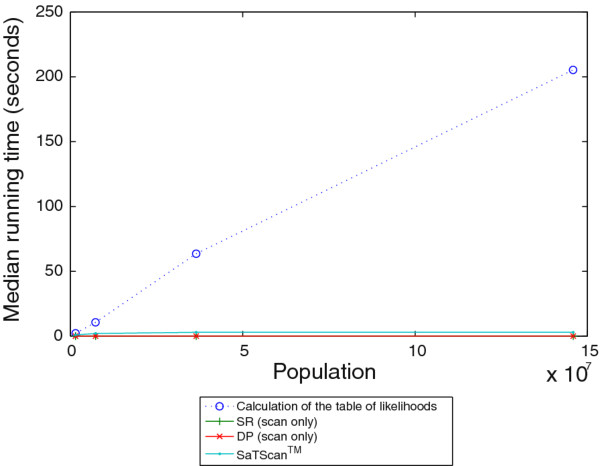
**Comparison of running time across a range of population sizes**. Comparison of running time for a 10 × 10 grid across populations of 1, 5, 25, and 100 times the original 2000 census population of Allegheny County. The plot shows the running time of the calculation of the table of likelihoods, the scan of the SR algorithm, the scan of the DP algorithm, and SaTScan™.

Figures 10 and 11 show median timing results for the likelihood calculation module, the scan modules of the SR and DP algorithms, and SaTScan™. All tests were performed on a desktop PC with a quad core 2.66 GHz processor and 4 GB of RAM. Figure 10 shows timing results for grids of size 10 × 10, 20 × 20, 50 × 50, and 100 × 100. We produced data with these varying grid sizes by dividing the same original map of Allegheny county into smaller cells to obtain "larger" grids (in terms of cell counts). The results are in line with our theoretical analysis that the runtime of the DP scan, like that of the SR scan, scales as a square of the number of cells in the grid. The results also show that grid size does not appreciably affect the time taken to compute the table of likelihoods that the DP algorithm uses. It can be seen that SaTScan™ takes approximately five minutes to complete a scan on a 50 × 50 grid, while the DP algorithm can complete a scan on a 100 × 100 grid in the same amount of time. A scan on a 100 × 100 grid by SaTScan™would take on the order of hours (not shown). The longer runtimes incurred by SaTScan™ for larger size grids are largely influenced by the 999-fold randomization testing that SaTScan™ uses to compute *p*-values for the scan statistic. The same observation was made by Neill, Moore, and Cooper in previous work [13].

Figure 11 shows timing results for 1, 5, 25, and 100 times the original census population of Allegheny County. We produced data with these varying population sizes by merely counting each individual multiple times. The results are consistent with our theoretical analysis that the likelihood calculation phase of the implementation scales linearly with the population size due to the agent-based nature of our model. The DP and SR scan times are not affected by variations in populations size, since those algorithms always query likelihoods for entire rectangles. The runtime of SaTScan™ is not appreciably affected by changes in the population size, which can be explained by the fact that as a count-based method, it deals with per-location counts.

## Conclusions

This paper described a dynamic programming algorithm for spatial cluster detection. The algorithm specifies alternative clustering hypotheses in terms of colored tilings of the surveillance grid. We showed how the algorithm can be used both for characterizing the location and shape of the most likely clusters and for calculating the posterior probability of the presence of clusters in the data.

Tests of general detection in terms of area under the ROC curve show that the new method is not statistically significantly better than the naïve calculation that only averages over single-rectangle hypotheses. This result appears to remain the case even when the true outbreaks cannot be reasonably approximated by a single rectangle. We conjecture that the additive nature of the process of model averaging either gives the naïve method more detection power than expected or hinders the dynamic programming method. On the one hand, even though the single-rectangle method cannot accurately capture multiple clusters with a single hypothesis, it can accurately capture parts of the multi-cluster scenario (the single rectangles that compose a multi-rectangle scenario). These partial agreements may make a sufficient contribution to the likelihoods so that averaging over all hypotheses results in relatively high posterior probability for the multi-cluster scenario. On the other hand, while the dynamic programming algorithm is able to accurately capture a multi-cluster scenario in a single hypothesis, it does consider an exponential number of alternative hypotheses that do not accurately describe the outbreak scenario. It is possible that when averaging over the entire hypothesis space the score of the accurate hypotheses is simply outweighed by the alternative scores. The reasons for why the two methods perform similarly may be a combination of both of these reasons and possibly others. Further investigation is needed to explain these results fully.

In spite of the lack of improvement in general detection power, tests of location and shape characterization show that under certain conditions the dynamic programming method performs better than the naïve SR method and the greedy method. The most notable improvement is in the precision of cluster locations detected, as DP algorithm tends to output fewer false-positive outbreak cells than the other algorithms. Recall of cluster locations is on par with the greedy method.

Measurements of the DP algorithm's running time over varying grid and population sizes showed that it scales well to larger grids, being able to complete a scan of a 100 × 100 grid in about five minutes. The measurements also show that the dynamic programming algorithm has a general advantage of taking no more time than the naïve single-rectangle scan. Since the scan itself relies on having access to a likelihood function for the likelihood of an outbreak in a particular rectangle, the runtime will depend on nature of the likelihood calculation. In our implementation and using our particular disease model, this calculation could be performed in a separate module and timed separately. We observed that the likelihood calculation scales linearly with the population using our agent-based model, taking under four minutes for a population of almost 150 million.

Just like the baseline methods considered, the dynamic programming algorithm can be applied to other likelihood models as long as they provide a likelihood as a function of location. In particular, this means that even though an agent-based model was used in the experiments, these methods are also suited for use with a count-based model. These methods can also be extended to additional space dimensions as well as time, and multiple cluster classes (outbreak states). The DP algorithm can be also modified to perform finer characterization such as finding the most likely values of *F *and *K *associated with each tile. By extension, using a different underlying model the algorithm could be modified to estimate the most likely values for that model's parameters. In that sense, there are many potential applications for the dynamic programming algorithm, as well as many potential avenues for further investigation.

## Endnotes

^a^SaTScan™ is a trademark of Martin Kulldorff. The SaTScan™ software was developed under the joint auspices of (i) Martin Kulldorff, (iii) the National Cancer Institute, and (iii) Farzad Mostashari of the New York City Department of Health and Mental Hygiene.

## Competing interests

The authors declare that they have no competing interests.

## Authors' contributions

GF Cooper conceived of the basic dynamic programming algorithm presented here, advised and supervised all stages of research, and participated in the drafting of the manuscript. X Jiang performed initial tests and initial implementation of the model selection version of the dynamic programming algorithm and performed the analysis of the number of tilings searched. Y Sverchkov contributed to the development of the model averaging version of the dynamic programming algorithm, implemented and tested all the algorithms presented in this paper, and took the lead in drafting this paper. All authors read and approved the final manuscript.

## Pre-publication history

The pre-publication history for this paper can be accessed here:

http://www.biomedcentral.com/1472-6947/12/22/prepub

## Supplementary Material

Additional file 1 **Appendix A: Tiling selection code**. Pseudocode of the tiling selection algorithm that returns the highest scoring tiling and its score. Click here for file

Additional file 2 **Appendix B: The number of tilings**. Calculation of the number of tilings considered by the algorithm. Click here for file

Additional file 3 **Appendix C: Normalization function**. Proof of equation (32). Click here for file
