# Supplementary material for: Spatial cluster detection using dynamic programming
Source: BMC Med Inform Decis Mak. 2012 Mar 25;12:22. doi: 10.1186/1472-6947-12-22 (PMC3403878; doi:10.1186/1472-6947-12-22)
Supplement: Additional file 1 — Appendix A: Tiling selection code. Pseudocode of the tiling selection algorithm that returns the highest scoring tiling and its score. [file 1472-6947-12-22-S1.PDF]

Appendix A: Tiling selection code

In order to track the highest scoring tiling along with its score, let us associate a set of tiles and a score in an ordered pair $s = (a, \mathcal{T})$. Here, a is the score and $\mathcal{T}$ is a (possibly partial) tiling, meaning that it is a set of non-overlapping tiles. When the tiling is partial, the tiles do not tile the entire space. The notation s_{score} and s_{tiling} will be used to refer to the elements of the ordered pair. The notation $T(x, R_L, R_H, C_L, C_H)$ will be used to represent a tile with outbreak state x (0 or 1) and boundaries R_L, R_H, C_L, C_H. $\emptyset$ denotes the empty set.

Tiling selection algorithm:

For a grid of size $R \times C$
Track best hypotheses: *bestR*, *bestC*
Keep caches *rCache*, *cCache*
Init:
    $rCache[0] \leftarrow (1, \emptyset)$
    $cCache[0] \leftarrow (1, \emptyset)$
for R_H **from** 1 **to** R
    $bestR \leftarrow (0, \emptyset)$
    for R_L **from** 1 **to** R_H
        for C_H **from** 1 **to** C
            $bestC \leftarrow (0, \emptyset)$
            for C_L **from** 1 **to** C_H
                $x \leftarrow \mathrm{argmax}_{x \in \{0,1\}}\, score(x, R_L, R_H, C_L, C_H)$
                $s \leftarrow (score(x, R_L, R_H, C_L, C_H), \{T(x, R_L, R_H, C_L, C_H)\})$
                if $bestC_{score} < cCache[C_L - 1]_{score} \cdot s_{score}$ **then**
                    $bestC \leftarrow (cCache[C_L - 1]_{score} \cdot s_{score}, cCache[C_L - 1]_{tiling} \cup s_{tiling})$
                end if
            end for
            $cCache[C_H] \leftarrow bestC$
        end for
        if $bestR_{score} < rCache[R_L - 1]_{score} \cdot bestC_{score}$ **then**
            $bestR \leftarrow (rCache[R_L - 1]_{score} \cdot bestC_{score}, rCache[R_L - 1]_{tiling} \cup bestC_{tiling})$
        end if
    end for
    $rCache[R_H] \leftarrow bestR$
end for
return *bestR*
