# Supplementary material for: Spatial cluster detection using dynamic programming
Source: BMC Med Inform Decis Mak. 2012 Mar 25;12:22. doi: 10.1186/1472-6947-12-22 (PMC3403878; doi:10.1186/1472-6947-12-22)
Supplement: Additional file 2 — Appendix B: The number of tilings. Calculation of the number of tilings considered by the algorithm. [file 1472-6947-12-22-S2.PDF]

Appendix B: The number of tilings

Below, we analyze the size of the hypothesis space scanned by the algorithm in relation to the size of the grid.

The one-dimensional case

In the one-dimensional case we are given a grid made up of one row and C columns. In the case where $C = 1$, we only have one cell in the grid, and we consider the two hypotheses of whether it is an outbreak cell or a non-outbreak cell. Let $V(C)$ represent the number of hypotheses considered for a row of C cells. In the case of $C > 1$, we consider whether the range of cells $C_L \ldots C$ is an outbreak or a non-outbreak rectangle for each of the $V(C_L - 1)$ tilings of the cells left of cell C_L[1].

Hence, the total number of hypotheses $V(C)$ investigated by the algorithm is given by the following recurrence:

$$\begin{aligned} V(1) &= 2 \\ V(C) &= 2 + 2V(1) + 2V(2) + \ldots + 2V(C-1) \\ &= V(C-1) + 2V(C-1) \\ &= 3V(C-1). \end{aligned}$$

The solution to this recurrence is

$$V(C) = 2 \times 3^{C-1}. \tag{1}$$

Notice that this number includes many non-outbreak hypotheses. For example, as we can see from Figure 3, there are two non-outbreak hypotheses when $C = 2$ out of a total of 6 hypotheses. Following a similar line of reasoning as above, when we only consider non-outbreak rectangles, the total number $W(C)$ of non-outbreak hypotheses is given by this recurrence:

$$\begin{aligned} W(1) &= 1 \\ W(C) &= 1 + W(1) + W(2) + \ldots + W(C-1) \\ &= W(C-1) + W(C-1) \\ &= 2W(C-1). \end{aligned}$$

The solution to this recurrence is

$$W(C) = 2^{C-1} \tag{2}$$

Then, the total number of outbreak hypotheses is given by

$$V(C) - W(C) = 2 \times 3^{C-1} - 2^{C-1}$$

The two-dimensional case

In the two-dimensional case we are given an $R \times C$ grid of cells.

Let

$$l = 2 \times 3^{C-1}.$$

From Equation 1, we see that the number $V(R, C)$ of hypotheses investigated by the algorithm is given by the following recurrence:

$$\begin{aligned} V(1, C) &= l \qquad (3) \\ V(R, C) &= l + lV(1, C) + lV(2, C) + \ldots + lV(R-1, C) \\ &= V(R-1, C) + lV(R-1, C) \\ &= (l+1)V(R-1, C). \end{aligned}$$

[1] If $C_L = 1$ then there are no cells left of C_L and the number of tilings of an empty set of cells is vacuously 1, hence, the recurrence can be alternatively defined as statring with $V(0) = 1$.

The solution to this recurrence is

$$V(R,C) = (l+1)^{R-1}l.$$

Substituting for l, we have that the total number of hypotheses is

$$V(R,C) = (2 \times 3^{C-1} + 1)^{R-1} \times 2 \times 3^{C-1}. \tag{4}$$

To determine the number $W(R,C)$ of non-outbreak hypotheses, we let

$$l = 2^{C-1},$$

due to Equation 2, then the total number of non-outbreak hypotheses is given by recurrence 3 with W replacing V, which has the solution

$$W(R,C) = (l+1)^{R-1}l.$$

Substituting for l, we have that the total number of no-outbreak hypotheses is

$$W(R,C) = (2^{C-1} + 1)^{R-1} \times 2^{C-1}. \tag{5}$$

Therefore, the total number of outbreak hypotheses is equal to

$$V(R,C) - W(R,C) = (2 \times 3^{C-1} + 1)^{R-1} \times 2 \times 3^{C-1} - (2^{C-1} + 1)^{R-1} \times 2^{C-1}. \tag{6}$$
