# Supplementary material for: Spatial cluster detection using dynamic programming
Source: BMC Med Inform Decis Mak. 2012 Mar 25;12:22. doi: 10.1186/1472-6947-12-22 (PMC3403878; doi:10.1186/1472-6947-12-22)
Supplement: Additional file 3 — Appendix C: Normalization function. Proof of equation (32). [file 1472-6947-12-22-S3.PDF]

Appendix C: Normalization function

The claim we wish to prove is that given an $R \times C$ grid and the dynamic programming algorithm for summation over tilings given in Figure 8, when the score is constant, i.e., $h(R_L, R_H, C_L, C_H) = y$ for some fixed value y, the value returned by the algorithm is

$$f(R, C, y) = y(1+y)^{C-1}\left(1 + y(1+y)^{C-1}\right)^{R-1} \tag{1}$$

Proof. When $h(R_L, R_H, C_L, C_H)$ is equal to a constant y it is independent of its four parameters R_L, R_H, C_L, C_H, and consequently the result given by the inner two loops (lines 10 to 17) is independent of R_L and R_H. Thus we can treat the inner pair of nested loops and outer pair of nested loops independently, meaning that the value returned by the entire tiling summation algorithm is equal to:

$$g(R, g(C, y)) \tag{2}$$

where $g(n, x)$ is the value returned by just one pair of nested loops, namely:

```
1   Function g(n, x):
2   Keep a running total: total ← 0
3   Keep a cache: cache
4   Init: cache[0] ← 1

5   for i from 1 to n
6       total ← 0
7       for j from 1 to i
8           total ← total + cache[j − 1] · x
9       end for
10      cache[i] ← total
11  end for
12  return total
```

Note that since *total* is reset to zero for each i (line 6), and since *cache*[0] is always 1, the value of *total* at a particular value of j is independent of the value of i, moreover the value of *cache*$[j-1]$ is always exactly the value of *total* at the end of the inner loop when $i = j - 1$ (due to line 10), hence an equivalent calculation is:

```
1   Second implementation of g(n, x):
2   Keep a running total: total ← 0
3   Keep a cache: cache
4   Init: cache[0] ← 1

5   for j from 1 to n
6       total ← total + cache[j − 1] · x
7       cache[j] ← total
8   end for
9   return total
```

At this point we can prove by induction that $g(n, x) = x(1+x)^{n-1}$:

Basic case: for $n = 1$ the loop completes one iteration in which it sets

$$total = 0 + cache[0] \cdot x = x = x(1+x)^{1-1} \tag{3}$$

Inductive case: given that $g(n-1,x) = x(1+x)^{n-2}$ when running the algorithm for $g(x,n)$ at the end of the $n-1$st iteration

$$cache[n-1] = total = x(1+x)^{n-2} \tag{4}$$

by the inductive hypothesis. The nth iteration sets

$$total = x(1+x)^{n-2} + x(1+x)^{n-2} \cdot x = x(1+x)^{n-2} \cdot (1+x) = x(1+x)^{n-1} \tag{5}$$

This proves that $g(n,x) = x(1+x)^{n-1}$, and consequently, the value returned by the full dynamic programming algorithm must be

$$f(R,C,y) = g(R, g(C,y)) = y(1+y)^{C-1}\left(1 + y(1+y)^{C-1}\right)^{R-1} \tag{6}$$

□
